# Low Levels of IgM Recognizing 4-Hydroxy-2-Nonenal-Modified Apolipoprotein A-I Peptide and Its Association with the Severity of Coronary Artery Disease in Taiwanese Patients

**DOI:** 10.3390/cimb46060374

**Published:** 2024-06-20

**Authors:** Meng-Huan Lei, Po-Wen Hsu, Yin-Tai Tsai, Chen-Chi Chang, I-Jung Tsai, Hung Hsu, Ming-Hui Cheng, Ying-Li Huang, Hung-Tse Lin, Yu-Cheng Hsu, Ching-Yu Lin

**Affiliations:** 1 Cardiovascular Center, Lo-Hsu Medical Foundation Luodong Poh-Ai Hospital, Yilan 26546, Taiwan; mhlei6401@yahoo.com.tw; 2 Preventive Medical Center, Lo-Hsu Medical Foundation Luodong Poh-Ai Hospital, Yilan 26546, Taiwan; sh46913.sh46913@msa.hinet.net; 3 Department of Medicine Laboratory, Shuang Ho Hospital, Taipei Medical University, New Taipei City 23561, Taiwan; 08826@s.tmu.edu.tw; 4 Department of Laboratory Medicine, Taipei City Hospital Heping-Fuyou Branch, Taipei 10027, Taiwan; a0679@tpech.gov.tw; 5 Ph.D. Program in Medical Biotechnology, College of Medical Science and Technology, Taipei Medical University, Taipei 11031, Taiwan; d609108005@tmu.edu.tw (I.-J.T.); 91a018@gmail.com (M.-H.C.); 6 Medical Quality Department, Lo-Hsu Medical Foundation Luodong Poh-Ai Hospital, Yilan 26546, Taiwan; itisbill1209@hotmail.com; 7 Department of Laboratory Medicine, Lo-Hsu Medical Foundation Luodong Poh-Ai Hospital, Yilan 26546, Taiwan; 8 Section of Laboratory, Lo-Hsu Medical Foundation Luodong Poh-Ai Hospital, Yilan 26546, Taiwan; daodai38@gmail.com; 9 Department of Laboratory Medicine, LinKou Chang Gung Memorial Hospital, Taoyuan 33305, Taiwan; timbaseball@cgmh.org.tw; 10 Department of Medical Biotechnology and Laboratory Science, College of Medicine, Chang Gung University, Taoyuan 33302, Taiwan; 11 School of Medical Laboratory Science and Biotechnology, College of Medical Science and Technology, Taipei Medical University, Taipei 11031, Taiwan

**Keywords:** coronary artery disease, apolipoprotein A-I, 4-hydroxy-2-nonenal, autoantibody, plasma, oxidative stress

## Abstract

Autoantibodies against apolipoprotein A-I (ApoA-I) are associated with cardiovascular disease risks. We aimed to examine the 4-hydroxy-2-nonenal (HNE) modification of ApoA-I in coronary artery disease (CAD) and evaluate the potential risk of autoantibodies against their unmodified and HNE-modified peptides. We assessed plasma levels of ApoA-I, HNE-protein adducts, and autoantibodies against unmodified and HNE-peptide adducts, and significant correlations and odds ratios (ORs) were examined. Two novel CAD-specific HNE-peptide adducts, ApoA-I^251–262^ and ApoA-I^70–83^, were identified. Notably, immunoglobulin G (IgG) anti-ApoA-I^251–262^ HNE, IgM anti-ApoA-I^70–83^ HNE, IgG anti-ApoA-I^251–262^, IgG anti-ApoA-I^70–83^, and HNE-protein adducts were significantly correlated with triglycerides, creatinine, or high-density lipoprotein in CAD with various degrees of stenosis (<30% or >70%). The HNE-protein adduct (OR = 2.208-fold, *p* = 0.020) and IgM anti-ApoA-I^251–262^ HNE (2.046-fold, *p* = 0.035) showed an increased risk of progression from >30% stenosis in CAD. HNE-protein adducts and IgM anti-ApoA-I^251–262^ HNE may increase the severity of CAD at high and low levels, respectively.

## 1. Introduction

Plaque buildup inside coronary arteries, which results in coronary artery stenosis, is the primary cause of coronary artery diseases (CADs) [1]. CADs are the third-leading cause of mortality worldwide [2]. In 2019, cardiovascular disease (CVD) was Taiwan’s second-leading cause of death, with over 17,000 people dying each year from CAD [3]. In epidemiological studies, family history, age, gender, ethnicity, diabetes, hypertension, hyperlipidemia, smoking, and obesity were all identified as risk factors for CAD [4].

Roles of apolipoprotein A-I (ApoA-I) include reverse cholesterol transport (RCT) back to low-density lipoprotein (LDL) or toward excretion via the liver, as well as the conservation of endothelial function [5]. High-density lipoprotein (HDL), in which ApoA-I is the predominant constituent, may play a protective role in preventing the generation of oxidatively modified LDL in atherogenesis [6]. Burger and Dayer reported that HDL-associated ApoA-I is a negative acute-phase protein, the level of which decreases during the acute phase [7]. Compared to patients with myocardial infarction, unstable angina, and stable angina, ApoA-I levels were significantly higher in subjects with controls [8,9]. Approximately 20% of individuals in the general population exhibit heightened levels of anti-ApoA-I immunoglobulin G (IgG), which have been independently linked to a higher risk of cardiovascular disease [10,11]. Patients with myocardial infarction and unstable angina have elevated anti-ApoA-I IgG levels [11,12], which can reduce HDL antioxidant activity [13].

Oxidative stress can aggravate the lipid peroxidation process through reactive oxygen species attacking omega-6 polyunsaturated fatty acids, which causes LDL alterations, the oxidization of which is a key component of atherogenesis [14]. A 4-hydroxy-2-nonenal (HNE)-modified LDL (HNE-LDL) may have significant impacts on the development of atherosclerosis [15]. HNE, a byproduct of lipid peroxidation reactions, can interact with amino acid residues in proteins to form either Michael adducts or Schiff base adducts [16]. The leucine (L), lysine (K), histidine (H), glutamine (Q), cysteine (C), alanine (A), and arginine (R) of HNE-amino acid Michael adducts experience an increase in mass of 156 Da, in which the alkene bond in HNE interacts with particular amino acid residues in the protein. Moreover, the amino acid residues (CHKAL) of HNE-amino acid Schiff base adducts experience an increase in mass of 138 Da, where the aldehyde group of HNE attacks amino acid residues in the proteins [17,18,19]. HNE is a type of oxidation-specific epitope (OSE) that is recognized by the innate immune system, particularly by natural immunoglobulin M (IgM) antibodies [20]. Furthermore, HNE is a proatherogenic agent and promotes plaque advancement [21]. The HNE-modified protein (HNE-protein) adduct, a biomarker of oxidative stress, is an autoantigen that has neo-epitope characteristics that elicit specific autoantibodies [22]. Autoantibodies were observed to recognize HNE-derived epitopes in various diseases, such as CAD, Alzheimer’s disease, alcoholic liver disease, systemic lupus erythematosus, and Sjögren’s syndrome [23,24,25,26,27]. Autoantibodies specifically targeting HNE-LDL can be detected in atherosclerotic plaques [28].

In the present study, our aim was to investigate the levels of ApoA-I, HNE-protein adduct-presented oxidative stress, and autoantibodies against ApoA-I peptides and HNE-ApoA-I peptides in the progression of CAD severity. In addition, we strove to understand whether the epitope of the ApoA-I and HNE-ApoA-I peptides produced autoantibodies. Furthermore, we assessed the potential protective or increasing risk effects of HNE-protein adducts and autoantibodies in the development of >30% stenosis in CAD.

## 2. Materials and Methods

### 2.1. Study Population

In total, 272 plasma samples and data from 212 patients with CAD and 60 age- and sex-matched healthy controls (HCs) were collected from the Cardiovascular Center and Department of Laboratory Medicine, Lo-Hsu Medical Foundation Luodong Poh-Ai Hospital (Yilan, Taiwan) from 1 September 2018 to 31 October 2019, and these were divided into a discovery set (60 samples) and a validation set (212 samples). Inclusion criteria included (1) patients aged 20~80 years old; (2) a cardiologist performed quantitative coronary angiography on patients with CAD and found at least one major epicardial vessel with >50% diameter stenosis that confirmed coronary artery stenosis, and (3) angina-free subjects. Exclusion criteria included (1) subjects with mental disorders or disturbances of consciousness and those who could not understand the statements of the research team, (2) patients with acute coronary syndrome, (3) patients with a history of abnormal blood coagulation, and (4) patients who were unwilling to participate in this study. Coronary artery stenosis was calculated using the following equation: (1 − (the diameter of diseased vessel/the diameter of reference)) × 100% according to the result of quantitative coronary angiography [29]. Depending on the severity of coronary artery stenosis, CAD sufferers were divided into three groups: <30%; 30%~70%; and >70%. In the discovery set, 20 HCs (14 male and 6 female patients, aged 34 ± 3.39 years), 20 CAD patients with ≤50% stenosis (14 male and 6 female patients, aged 63.6 ± 9.36 years), and 20 CAD patients with >50% stenosis (14 male and 6 female patients, aged 63.4 ± 8.38 years) were included. In the validation set, 40 HCs (24 male and 16 female patients, aged 38.3 ± 10.19 years), 46 CAD patients with <30% stenosis (31 male and 15 female patients, aged 62.93 ± 10.22 years), 47 CAD patients with 30%~70% stenosis (33 male and 14 female patients, aged 63.06 ± 9.86 years), and 79 CAD patients with >70% stenosis (60 male and 19 female patients, aged 62.43 ± 9.07 years) were included.

### 2.2. Study Protocol

**Figure 1:**
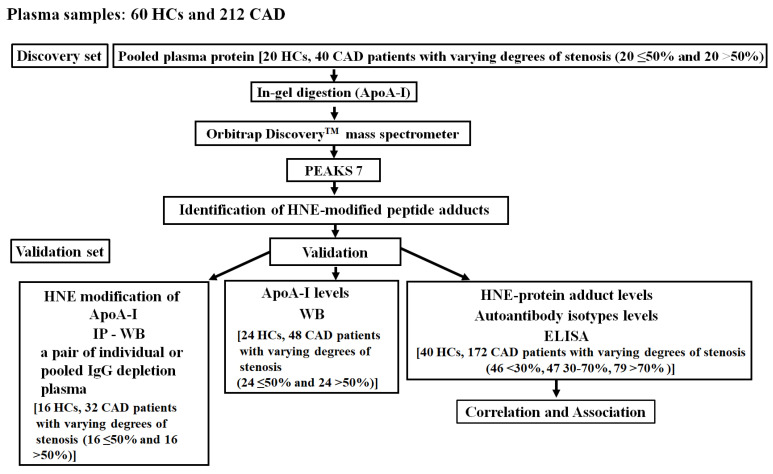
Flow chart. HC, healthy control; CAD, coronary artery disease; ApoA-I, apolipoprotein A-I; HNE, 4-hydroxy-2-nonenal; IP, immunoprecipitation; WB, Western blotting; HNE-protein adduct, HNE-modified protein adduct; ELISA, enzyme-linked immunosorbent assay.

A flowchart of this study is shown in Figure 1. In the discovery set, novel HNE-peptide adducts derived from ApoA-I were discovered using 20 pairs of pooled plasma samples, one-dimensional sodium dodecyl sulfate–polyacrylamide gel electrophoresis (1D SDS-PAGE), in-gel digestion, nano-liquid chromatography–tandem mass spectrometry (nano-LC-MS/MS), and PEAKS 7 software (Bioinformatics Solutions, Waterloo, OA, Canada). For the validation set, we used immunoprecipitation (IP)–Western blotting (WB) to confirm the HNE modifications of ApoA-I and took a pair of individual plasma samples or randomly from another 15 pairs of pooled IgG-depleted plasma of HCs versus CAD patients with various levels of stenosis (≤50% and >50%). WB was applied to 24 pairs of plasma samples from HCs and those from CAD patients with various levels of stenosis (≤50% and >50%) to measure ApoA-I levels. An enzyme-linked immunosorbent assay (ELISA) was used to measure HNE-protein adducts, as well as anti-unmodified and anti-HNE-ApoA-I peptide adduct autoantibody isotype levels in 40 HCs and 172 CAD patients with varying levels of stenosis (<30%, 30%~70%, and >70%). Moreover, correlations of HNE-protein adduct and autoantibody levels from blood tests were determined in CAD patients with various levels of stenosis. Next, associations between HNE-protein adducts and autoantibodies in CAD patients with >30% stenosis and risk of disease development were evaluated in comparison with CAD patients with <30% stenosis. Neglia et al. reported that significant CAD could be characterized by invasive coronary angiography as >50% left main stem stenosis, >70% major coronary artery stenosis, or 30% to 70% with fractional flow reserve ≤0.8 [30]. Moladoust et al. defined CAD with significant stenosis as > 70% stenosis and moderate stenosis as stenosis between 50% and 70% [31]. Furthermore, Hung and Cherng’s definition of CAD with <30% stenosis is the lack of a clinically relevant level of narrowing in the arteries [32]. Ong et al. indicated that >30% stenosis significantly affected the progression of carotid artery stenosis [29]. Thus, samples were categorized as CAD with <30% stenosis and CAD with <30% stenosis. The sample size (*n* = 86) of CAD patients with <30% stenosis included both HCs (*n* = 40) and CAD patients with <30% stenosis (*n* = 46). The potential sample size estimation was also considered. We calculated the sample size based on the mean and standard deviation (SD) of CAD patients with <30% stenosis and those with >30% stenosis (Supplementary Table S2). Power analysis is essential when using limited sample sizes; therefore, plasma samples were frozen at −80 °C until the experiment. The institutional review board of Cathay General Hospital approved this study, and all volunteers gave their informed consent to participate (CGH-LP106001).

### 2.3. In-Gel Digestion and Identification of Novel HNE Modifications Using Nano-LC-MS/MS and PEAKS 7 Software

**Figure 2:**
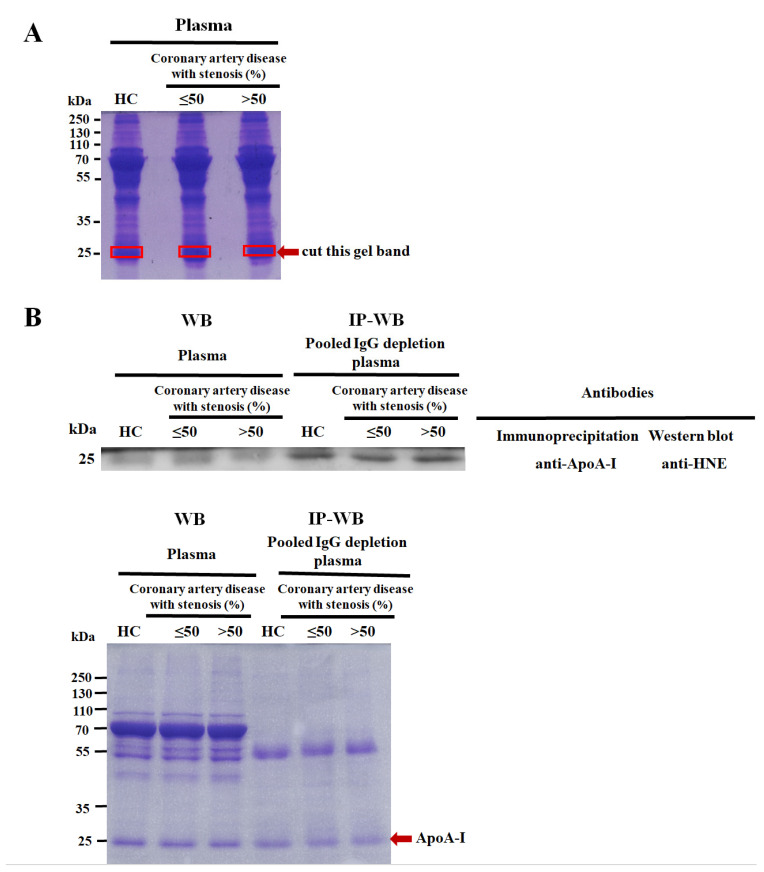
Confirmation of 4-hydroxy-2-nonenal (HNE)-modified apolipoprotein A-I (ApoA-I). (**A**) The percentage of SDS-PAGE gel and loading amounts of pooled plasma proteins were 10% and 50 µg, respectively, from 20 pairs of healthy controls (HCs) versus coronary artery disease (CAD) patients with various levels of stenosis (≤50% and >50%). The gel was stained with Coomassie brilliant blue (CBB) and cut into pieces at molecular weights of 24~26 kDa, as shown by the red arrow. (**B**) HNE-modified ApoA-I was validated using immunoprecipitation (IP)–Western blotting (WB). ApoA-I was immunoprecipitated with an anti-ApoA-I monoclonal antibody and 200 µg of pooled IgG-depleted plasma, using another 15 pairs of HCs and CAD patients with various levels of stenosis (≤50% and >50%). Then, a 10% SDS-PAGE gel and 5 µg protein were subjected to WB using an anti-HNE polyclonal antibody (upper panel). A pair of individually selected random plasma samples (HCs and CAD patients with various levels of stenosis of ≤50% and >50%) were used as controls, simultaneously used in IP-WB. A duplicate gel was stained with CBB as a loading control (bottom panel). The red arrow indicates the ApoA-I protein.

A Pierce™ Coomassie Plus (Bradford) Assay Kit (ThermoFisher Scientific, Waltham, MA, USA) was used to determine the total plasma protein level, and the protocol followed directions from the manufacturer. Fifty micrograms of pooled plasma protein samples from HCs versus CAD patients with various levels of stenosis (≤50% and >50%) were separated using 10% 1D SDS-PAGE (Hoefer^®^, Holliston, MA, USA), as per the Laemmli method [33]. The entire gel was stained with Coomassie brilliant blue (CBB) staining solution (Bio-Rad Laboratories, Hercules, CA, USA), and the gel band was incised to correspond to molecular weights of 24~26 kDa (Figure 2A). In-gel digestion was performed twice following the protocol of Uen et al. [34]. Next, tryptic peptide mixtures were infused into NanoLC-nanoESi-MS/MS, which was conducted using a nanoAcquity system (Waters, Milford, MA, USA) linked to an LTQ-Orbitrap XL^TM^ hybrid mass spectrometer (ThermoFisher Scientific, Bremen, Germany) equipped with a nanospray interface (Proxeon, Odense, Denmark). Novel HNE-peptide sequences and sites of plasma ApoA-I were identified using de novo peptide sequencing and the PeaksPTM module of PEAKS 7 software (Bioinformatics Solutions, Waterloo, OA, Canada). Further details are presented in the “Supplementary Information”.

### 2.4. Confirmation of HNE-Modified ApoA-I by IP-WB

The ApoA-I was captured with Protein A SepharoseTM CL-4B beads (GE Healthcare Bio-Sciences, Pittsburgh, PA, USA) coupled to a mouse anti-ApoA-I monoclonal antibody (H00000335-M01, Abnova, Taipei, Taiwan). After washing the beads, 5 μg immunoprecipitated ApoA-I was separated on 10% SDS-PAGE gels and transferred onto a polyvinylidene difluoride (PVDF) membrane (GE Healthcare Life Sciences, Piscataway, NJ, USA). The HNE modification of ApoA-I was confirmed using a goat polyclonal anti-HNE antibody (MBS536107, MyBioSource, San Diego, CA, USA) and horseradish peroxidase (HRP)-mouse conjugated anti-goat monoclonal IgG antibody (sc-2354, Santa Cruz Biotechnology) that served as the secondary antibody. The Luminata^TM^ Forte Western HRP substrate (Millipore Corporation, Billerica, MA, USA) was used to present the protein bands. As a loading control, another duplicate protein gel was stained with a CBB staining solution (Bio-Rad Laboratories). Details are presented in “Supplementary Information”.

### 2.5. Evaluation of ApoA-I Levels by WB

Plasma protein levels of ApoA-I were determined with a WB assay. ApoA-I (5 μg of protein in 10% SDS-PAGE) was evaluated using a mouse anti-ApoA-I monoclonal antibody (H00000335-M01, Abnova) and goat anti-mouse IgG-HRP (sc-2055, Santa Cruz Biotechnology). The band was detected using Millipore’s Western HRP Substrate. The blot densitometric measurement was performed with an ImageQuant 400TM Imager (GE Healthcare Life Sciences) and ImageJ software vers. 1.52t (National Institutes of Health, Bethesda, MD, USA). A duplicate protein gel was used to stain a CBB staining solution (Bio-Rad Laboratories) as a loading control. Details are presented in the “Supplementary Information”.

### 2.6. Quantification of Plasma HNE-Protein Adducts and Autoantibodies Recognizing Unmodified and HNE-ApoA-I Peptide Adducts by an ELISA

An ELISA was used to measure HNE-protein adduct levels following the method of Weber et al. [35]. Polypeptides corresponding to the 70~83 and 251~262 amino acid sequences of human ApoA-I, i.e., LLDNWDSVTSTFSK (named ApoA-I^70–83^) and VSFLSALEEYTK (named ApoA-I^251–262^), respectively, were synthesized (Yao-Hong Biotechnology, New Taipei City, Taiwan). Autoantibody isotype levels were measured using Liao’s ELISA method [36]. ApoA-I^70–83^ and ApoA-I^251–262^ were modified with HNE (CAS 75899-68-2, Millipore, Darmstadt, Germany) and labeled ApoA-I^70–83^ HNE and ApoA-I^251–262^ HNE, respectively [37]. Using an ELISA, IgG or IgM anti-ApoA-I and anti-HNE-ApoA-I peptide adduct autoantibodies were measured in the validation set (212 plasma samples), and all samples were tested twice. The optical density (OD) was measured at 450–620 nm. The numerical conversion of µg/mL through OD values was measured from a standard curve on each plate. Further details are presented in the “Supplementary Information”.

### 2.7. Statistical Analysis

Values of age, blood test parameters, ApoA-I, autoantibodies, and HNE-protein adducts are shown as the mean ± standard deviation (SD). We used the Kolmogorov–Smirnov test to assess data on HNE-protein adducts, autoantibodies, ApoA-I, and blood test parameters. HNE-protein adducts and autoantibodies had a non-normal distribution, which required a further nonparametric analysis. The Mann–Whitney U-test was used to analyze differences between the two groups, and the Kruskal–Wallis test was used to compare multiple groups. A Student’s *t*-test was used to evaluate the significance of blot densitometric quantification for ApoA-I and blood test levels. A Kruskal–Wallis test was used to compare levels of HNE-protein adducts, autoantibodies that recognized unmodified and HNE-peptide adducts between HCs, and CAD patients with various levels of stenosis (<30%, 30%~70%, >70%). Scheffe’s method was used to analyze the divergence between means for two groups. Then, a post hoc test with the Bonferroni correction was used for comparisons, adjusted to a significance level of 0.0167 for comparing three groups or a significance level of 0.0083 for comparing four groups. Spearman’s rank correlation coefficients [ρ (rho)] were used to evaluate correlations of autoantibodies and HNE-protein adducts with blood tests. A logistic regression was used to evaluate the association of HNE-protein adducts or autoantibodies causing a risk of disease progression between CAD patients with >30% stenosis versus <30% stenosis. After adjusting for age, gender, PCr, and HDL, multivariate-adjusted odds ratios (ORs) and their 95% CIs were calculated. The criterion value of the optimal area under the receiver operating characteristic curve (AUC) was the cutoff value. The test’s statistical significance level was set to *p* < 0.05. SAS vers. 9.3 (SAS Institute, Cary, NC, USA) was used to calculate the Student’s *t*-test, Mann–Whitney U-test, Kruskal–Wallis test, logistic regression, and correlations. GraphPad Prism vers. 5.0 (GraphPad Software, San Diego, CA, USA) was used to draw dot plots and correlation lines. A sample size estimate and power analysis were conducted using G*Power vers. 3.1 [38].

## 3. Results

### 3.1. Baseline Characteristics

The demographic, clinical, and laboratory characteristics of HCs and CAD patients are presented in Table 1. Age, number of smokers, rate of end-stage renal disease (ESRD), high blood pressure, usage of lipid-lowering drugs, HDL, triglycerides (TGs), creatinine (PCr), and HNE-protein adducts significantly differed between HCs and CAD patients (Table 1).

### 3.2. Identification and Validation of Novel HNE Modifications of Plasma ApoA-I

With the use of one-dimensional sodium dodecyl sulfate–polyacrylamide gel electrophoresis (1D SDS-PAGE), in-gel digestion, nano-liquid chromatography tandem mass spectroscopy (LC-MS/MS), PEAKS 7 software, and immunoprecipitation–Western blotting (IP-WB) to analyze pooled plasma samples (healthy controls (HCs) versus CAD patients with various levels of stenosis (≤50% and >50%), we identified and validated novel HNE modifications that were detected to have a molecular weight close to 25 kDa and denoted as ApoA-I (Figure 2, Table 2). Amino acid sequence coverage in ApoA-I was 90% (Supplementary Table S1). MS/MS spectra of three HNE-modified peptide (HNE-peptide) adducts in ApoA-I were acquired and are presented in Supplementary Figure S1. HNE modifications were identified through manual inspections. The HNE-peptide adducts, ^251^-VSFLSALEEYTK-^262^ (Supplementary Figure S1A), ^70^-LLDNWDSVTSTFSK-^83^ (Supplementary Figure S1B), and ^52^-DYVSQFEGSALGK-^64^ (Supplementary Figure S1C), were examined. Two HNE-peptide adducts, ApoA-I^251–262^ and ApoA-I^70–83^, were CAD-specific, and HNE Schiff base adducts experienced a 138.10446 Da shift in three residues (at K262, L70, and L71; Table 2). Another HNE-peptide adduct, ApoA-I^52–64^, was present in HCs and CAD patients and corresponded to a mass increase of 156.11504 Da (at Q56) with the HNE Michael adduct (Table 2).

### 3.3. Determination of Levels of Plasma in ApoA-I and HNE-Protein Adducts

In the discovery set, levels of plasma ApoA-I protein from HCs versus CAD patients with various levels of stenosis (≤50% and >50%) revealed no statistically significant difference (Supplementary Figure S2). In the validation set, levels of the plasma in the HNE-protein adducts of CAD patients with >70% stenosis (1.10-fold, *p* < 0.01) were significantly higher than levels in HCs (Table 1).

### 3.4. Determining Levels of Plasma Autoantibodies That Recognize Unmodified and HNE-ApoA-I Peptide Adducts

**Figure 3:**
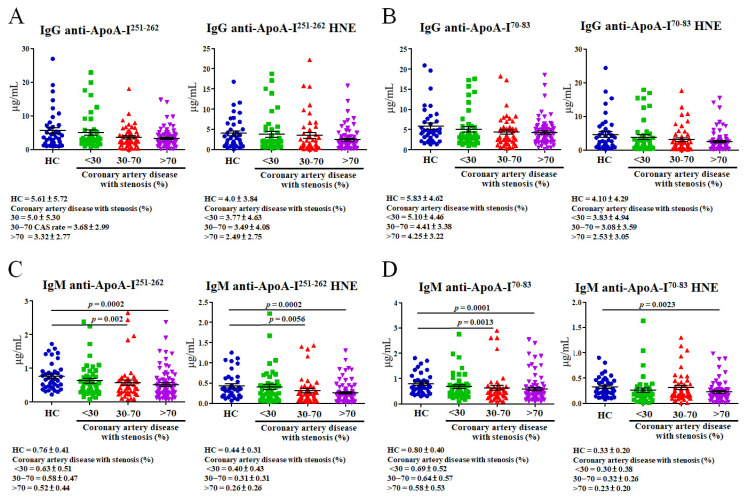
Levels of autoantibody isotypes against unmodified and 4-hydroxy-2-nonenal (HNE)-modified apolipoprotein A-I (ApoA-I) peptide adducts calculated using an ELISA are shown as dot plots. For the ELISA, plasma samples were taken from 40 healthy controls (HCs) and 142 coronary artery disease (CAD) patients with various levels of stenosis (46 with <30%, 47 with 30%–70%, and 79 with >70%). (**A**) IgG anti-ApoA-I^251–262^ and IgG anti-ApoA-I^251–262^ HNE. (**B**) IgG anti-ApoA-I^70–83^ and IgG anti-ApoA-I^70–83^ HNE. (**C**) IgM anti-ApoA-I^251–262^ and IgM anti-ApoA-I^251–262^ HNE. (**D**) IgM anti-ApoA-I^70–83^ and IgM anti-ApoA-I^70–83^ HNE.

An ELISA was used to detect autoantibodies that recognized ApoA-I peptides and HNE-ApoA-I peptide adducts. The levels of IgG for anti-ApoA-I^251–262^, anti-ApoA-I^251–262^ HNE, anti-ApoA-I^70–83^, and anti-ApoA-I^70–83^ HNE did not significantly differ among CAD patients with various levels of stenosis (<30%, 30%~70%, and >70%) versus HCs (Figure 3A,B). The levels of IgM anti-ApoA-I^251–262^ were significantly lower in CAD patients compared to HCs with 30%~70% stenosis (by 1.31-fold; *p* = 0.002) and those with >70% stenosis (by 1.46-fold; *p* = 0.0002) (Figure 3C, left panel). IgM anti-ApoA-I^251–262^ HNE levels in CAD patients with 30%~70% stenosis (1.42-fold, *p* = 0.0056) and >70% stenosis (1.69-fold, *p* = 0.0002) were significantly lower than levels in HCs (Figure 3C, right panel). Levels of IgM anti-ApoA-I^70–83^ were significantly lower in CAD patients compared to HCs with 30%~70% stenosis (by 1.25-fold; *p* = 0.0013) and >70% stenosis (by 1.38-fold; *p* = 0.0001) (Figure 3D, left panel). IgM anti-ApoA-I^70–83^ HNE levels in CAD patients with >70% stenosis (1.43-fold; *p* = 0.0023) were significantly lower than levels in HCs (Figure 3D, right panel).

### 3.5. Correlations of Plasma HNE-Protein Adduct and Anti-Unmodified and Anti-HNE-Peptide Adduct Autoantibodies with Blood Tests in CAD Patients with Various Degrees of Stenosis

HNE-protein adduct and autoantibodies were measured using a nonlinear regression. Spearman’s correlations were used to estimate the relationships between autoantibodies and HNE-protein adducts with blood parameters, such as total cholesterol (T-CHOL), HDL, low-density lipoprotein (LDL), triglycerides (TGs), and creatinine (PCr).

**Figure 4:**
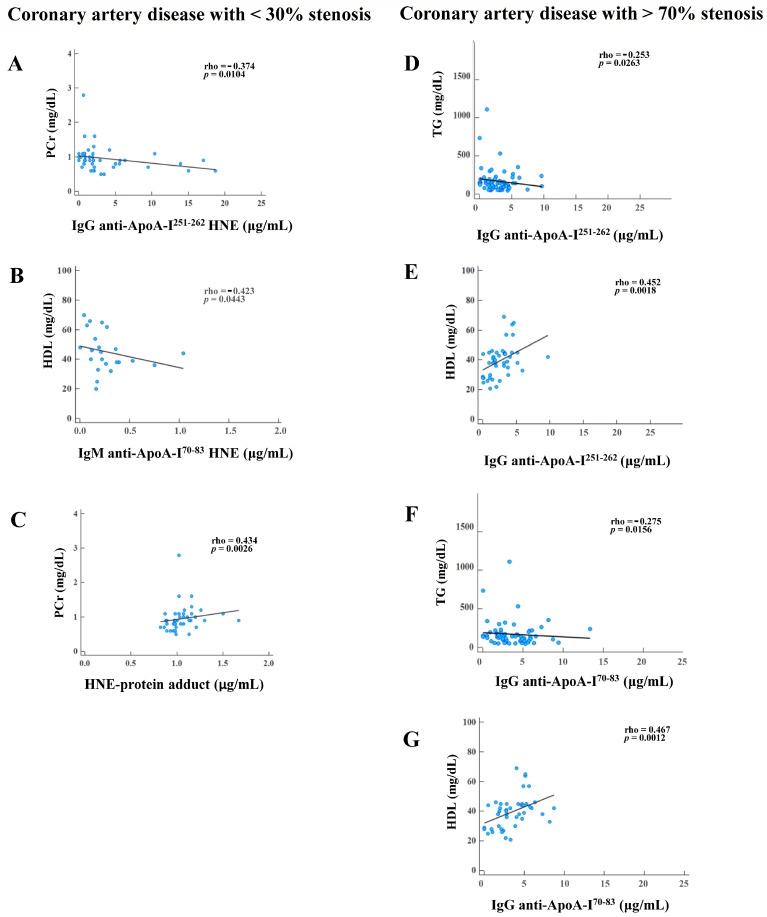
Results showing significant correlations of autoantibody isotypes against unmodified and 4-hydroxy-2-nonenal (HNE)-modified apolipoprotein A-I (ApoA-I) peptide adducts, HNE-protein adducts, and blood tests in coronary artery disease (CAD) patients with various degrees of stenosis (<30% and >70%). CAD with <30% stenosis: (**A**) creatinine (PCr) versus IgG anti-ApoA-I^251–262^ HNE. (**B**) High-density lipoprotein (HDL) versus IgM anti-ApoA-I^70–83^ HNE. (**C**) PCr versus the HNE-protein adduct. CAD with >70% stenosis. (**D**) Triglycerides (TGs) versus IgG anti-ApoA-I^251–262^. (**E**) HDL versus IgG anti-ApoA-I^251–262^. (**F**) TGs versus IgG anti-ApoA-I^70–83^. (**G**) HDL versus IgG anti-ApoA-I^70–83^.

In Figure 4, significant negative associations were identified: PCr versus IgG anti-ApoA-I^251–262^ HNE in CAD patients with <30% stenosis (rho = −0.374, *p* = 0.0104); HDL versus IgM anti-ApoA-I^70–83^ HNE in CAD patients with <30% stenosis (rho = −0.423, *p* = 0.0443); TGs versus IgG anti-ApoA-I^251–262^ in CAD patients with >70% stenosis (rho = −0.253, *p* = 0.0263); and TGs versus IgG anti-ApoA-I^70–83^ in CAD patients with >70% stenosis (rho = −0.275, *p* = 0.0156), as shown in Figure 4A, Figure 4B, Figure 4D, and Figure 4F, respectively. Otherwise, PCr versus the HNE-protein adduct in CAD patients with <30% stenosis (rho = 0.434, *p* = 0.0026), HDL versus IgG anti-ApoA-I^251–262^ in CAD patients with >70% stenosis (rho = 0.452, *p* = 0.0018), and HDL versus IgG anti-ApoA-I^70–83^ in CAD patients with >70% stenosis (rho = 0.476, *p* = 0.0012) were significantly positively correlated, as shown in Figure 4C, Figure 4E, and Figure 4G, respectively. All the data are shown in Supplementary Figure S3.

### 3.6. Associations between Plasma Autoantibodies and HNE-Protein Adducts of CAD Patients with >30% Versus <30% Stenosis

This association study defined CAD patients with <30% stenosis as a combination of HCs and CAD patients with <30% stenosis. CAD patients with <30% stenosis were categorized as a substantially lower risk group, while CAD patients with >30% stenosis were categorized as a comparably higher risk group. An age-, gender-, PCr-, and HDL-adjusted multivariate logistic regression analyses were conducted to assess the association between CAD patients with <30% versus >30% stenosis and to calculate ORs of levels of HNE-protein adducts and autoantibodies progressing in CAD disease. As a consequence, higher levels of HNE-protein adducts and lower levels of IgM anti-ApoA-I^251–262^ HNE, respectively, showed 2.208-fold (*p* = 0.020, power = 0.938) and 2.046-fold (*p* = 0.035, power = 0.881) higher risks between CAD patients with <30% versus >30% stenosis, indicating a significant difference after adjustments for age, gender, PCr, and HDL in the logistic regression analyses (Table 3). In cases where the power value was <0.7 or the risk for the development of >30% stenosis in CAD patients did not differ significantly from that of CAD patients with <30% stenosis, the results of the adjusted ORs were not considered for the levels of the following: IgG anti-ApoA-I^251–262^; IgG anti-ApoA-I^251–262^ HNE; IgG anti-ApoA-I^70–83^; IgG anti-ApoA-I^70–83^ HNE; IgM anti-ApoA-I^251–262^; IgM anti-ApoA-I^70–83^; and IgM anti-ApoA-I^70–83^ HNE (Table 3).

## 4. Discussion

This was a single-site study conducted in Northeastern Taiwan. We conducted the first Taiwanese study on ApoA-I, HNE-protein adducts, and autoantibodies against the ApoA-I and HNE-ApoA-I peptides associated with angina-free CAD patients.

Several studies on patients with acute coronary syndrome have found that ApoA1 levels nonsignificantly and significantly decreased as the severity of CAD increased [39,40]. In this study, compared to HCs, ApoA-I levels in CAD patients with >50% stenosis were 1.12-fold higher, and in CAD patients with ≤50% stenosis, they were 1.17-fold higher, as shown in Supplementary Figure S2. This result may appear illogical; however, Boekholdt et al. found that patients who took statins had higher ApoA-I levels [41]. Tian et al. reported that current non-smokers had higher ApoA-I levels than smokers [42]. In Table 1, CAD patients with >50% stenosis have a higher ratio of current smokers and lipid-lowering agents. WB and blot densitometry may perhaps explain this study’s ApoA-I level differences. No study has examined the ApoA-I level in angina-free CAD patients.

In this study, we found CAD-specific HNE modifications at positions L70, L71, and K262 in ApoA-I^70–83^ and ApoA-I^251–262^ (Table 2). The post-translation modification (PTM) of ApoA-I may trigger protein aggregation, which can harm the protein structure [43], and this may impact protein function. Szapacs et al. demonstrated that HNE modification at H186 of ApoA-I might have influenced protein functions and was implicated in the activation of lecithin–cholesterol acyltransferase (LCAT) [44]. Afonso and Spickett reported that modified ApoA-I may impact HDL functioning by altering the HDL proteome [28]. HNE-peptides possess neo-epitope properties that can form a specific autoantibody [22]. Studies of angina-free CAD patients with autoantibodies against ApoA-I or HNE-ApoA-I have never been conducted. Teixeira et al. reported that ApoA-I-derived peptides containing ApoA-I^165–206^ and ApoA-I^241–266^ epitopes possessed the properties of autoantigens, inducing the high titers of IgG anti-ApoA-I autoantibodies in myocardial infarction patients [12]. Pagano et al. indicated that the ApoA-I^74–108^ and ApoA-I^244–266^ epitopes could elicit high concentrations of IgG anti-ApoA-I autoantibodies in patients with a non-ST-segment elevation myocardial infarction [45]. We found that levels of IgG against ApoA-I peptides and HNE-ApoA-I peptides in CAD patients with 30%~70% and >70% stenosis were slightly lower than those in HCs and CAD patients with <30% stenosis (Figure 3A,B). In fact, CAD patients with angina-free blood developed IgG against ApoA-I peptide and HNE-ApoA-I peptide autoantibodies, but autoantibody levels did not differ statistically in terms of stenosis levels. Our estimation revealed a significant negative correlation between higher levels of PCr and lower IgG anti-ApoA-I^251–262^ HNE in CAD patients with <30% stenosis (Figure 4A). Bagheri et al. discovered that increased serum creatinine (SCr) implied more severe CAD [46]. CAD patients with different levels of stenosis (<30% and >70%) had significantly higher levels of PCr than HCs (Table 1). Furthermore, we observed significant negative correlations between higher levels of TGs and lower IgG anti-ApoA-I^251–262^ and IgG anti-ApoA-I^70–83^ in CAD patients with >70% stenosis (Figure 4D,F). Kajikawa et al. found that high serum TG levels raised the risk of initial major cardiovascular events in patients with CAD [47]. In Table 1, plasma TGs were significantly higher in CAD patients with >70% stenosis than in HCs. Faergeman et al. showed that patients taking statins that have slightly increased plasma TG levels are at risk of new cardiovascular events, and this could be a valuable indicator of risk. Therefore, doctors should assess the etiology of hypertriglyceridemia, lifestyle changes, and TG-lowering medication efficacy [48]. In CAD patients with >70% stenosis, lower HDL levels were linked to lower IgG anti-ApoA-I^251–262^ and IgG anti-ApoA-I^70–83^ levels (Figure 4E,G). Kosmas et al. showed that HDL had cardiovascular atheroprotective effects and that its levels inversely affected CAD risk [49]. Among CAD patients with <30%, 30%~70%, and >70% stenosis, lipid-lowering agents were taken at rates of 23.9%, 31.9%, and 70.8%, respectively (Table 1). However, plasma HDL was significantly lower in CAD patients with >70% stenosis than in HCs, even when 70.8% of lipid-lowering agents were used (Table 1). Thus, we speculated that low IgG anti-ApoA-I^251–262^ and IgG anti-ApoA-I^70–83^ levels might be a potential risk factor in more severe CAD. The adjusted ORs of IgG anti-ApoA-I^251–262^ and IgG anti-ApoA-I^70–83^ were not considered because the power was <0.7 or the adjusted ORs did not differ significantly between CAD patients with >30% stenosis and <30% stenosis in Table 3. This meant that IgG against ApoA-I peptide and HNE-ApoA-I peptide autoantibodies showed that the sustained immunological responses had lost their ability to neutralize autoantigen.

The levels of IgM against ApoA-I and HNE-ApoA-I peptides in CAD patients with 30%~70% and >70% stenosis were statistically significantly lower than HCs and CAD patients with <30% stenosis (Figure 3C,D). IgM against ApoA-I peptide and HNE-ApoA-I peptide autoantibodies represent the body’s immediate responses that can neutralize autoantigen. Binder observed that HNE-peptide adducts, such as OSEs with neo-epitopes, could trigger the production of natural autoantibodies (NAAs) [50]. Natural IgM autoantibodies (IgM-NAAs) can bind and neutralize OSEs to prevent the excessive accumulation of cellular debris, such as HNE-protein adducts [20]. Furthermore, the complement-mediated phagocytosis of apoptotic cells in vivo requires IgM [51]. The IgM-NAA-HNE complex is phagocytosed by macrophages in atherosclerotic lesions that are dependent on C1q-calreticulin-CD91 or mannose-binding lectin (MBL) and MBL receptors [52]. However, pattern recognition receptors (PRRs) recognize HNE-protein adducts that are not being properly removed as damage-associated molecular patterns (DAMPs) and, thus, trigger the body’s innate immune system, which causes sterile inflammation [53]. For example, a cellular PRR, human lectin-like oxidized LDL receptor-1 (LOX-1), can interact with HNE-protein adducts to activate extracellular-signal-regulated kinase 1/2 and nuclear factor-kappa B in human aortic endothelial cells [54]. This can lead to endothelial dysfunction and atherosclerosis. Furthermore, Tsai et al. observed that levels of IgM against the HNE-peptide adducts decreased as the stenosis of CAD increased from <30% to >70% [55]. In CAD patients with <30% stenosis, increased HDL was positively linked to lowered IgM anti-ApoA-I^70–83^ HNE levels (Figure 4B). This study revealed that IgM anti-ApoA-I^70–83^ HNE may offer short-term protection for CAD patients with <30% stenosis. However, it did not have a potential risk in the severity of CAD under a power of <0.7, nor did the adjusted ORs show any differences (Table 3). Furthermore, studies have shown that lower levels of IgM anti-ApoA-I^251–262^ HNE posed a significant risk factor for worsening >30% stenosis in CAD (Table 3). We found a strong positive correlation between elevated PCr and elevated HNE-protein adduct levels in CAD patients with <30% stenosis (Figure 4C). Verma et al. showed a notable correlation between blood creatinine levels and oxidative stress in those with end-stage renal disease (ESRD) [56]. Table 1 shows an increase in the prevalence of ESRD in CAD patients with various levels of stenosis (from 30% to >70%). The plasma HNE-protein adduct levels in CAD patients with >70% stenosis were significantly higher than those in HCs, as shown in Table 1. Higher HNE-protein adduct levels may significantly increase the risk of deteriorating >30% stenosis in CAD (Table 3). Therefore, we hypothesized that an increase in the HNE-protein adduct level would indicate an increase in oxidative stress during the progression of CAD, particularly with >30% stenosis. However, we found that there were insufficient levels of IgM anti-ApoA-I^251–262^ HNE to neutralize the HNE-protein adduct, which contributed to the severity of CAD. Thus, we inferred that reduced levels of IgM-NAAs against HNE-peptide adducts could not effectively eliminate OSEs from plasma and may cause a rise in CAD risk.

There are some limitations to our study. Firstly, it is a single-site study involving Taiwanese people living in Northeastern Taiwan. Secondly, the average age of HCs is significantly lower than that of patients. Thirdly, the limited sample size of this study is a significant issue, and a greater sample size would allow for further analysis of adjusted ORs. Cases with power values <0.7 were not considered, including IgG anti-ApoA-I^251–262^ HNE, IgG anti-ApoA-I^70–83^, IgG anti-ApoA-I^70–83^ HNE, IgM anti-ApoA-I^70–83^, and IgM anti-ApoA-I^70–83^ HNE.

## 5. Conclusions

In this study, we discovered two CAD-specific HNE-peptide adducts in HNE-ApoA-I and confirmed the modification of ApoA-I by HNE. Several blood tests, such as HDL, TGs, and PCr, had significant correlations with IgG anti-ApoA-I peptides, IgG anti-HNE-ApoA-I peptide adducts, and HNE-protein adducts in CAD patients with < 30% or > 70% stenosis. The risk of developing CAD with >30% stenosis increased with elevated levels of HNE-protein adducts and reduced levels of anti-ApoA-I^251–262^ HNE IgM antibodies. We concluded that high HNE-protein adduct and low IgM anti-ApoA-I^251–262^ HNE levels may increase the severity of CAD. A larger sample size is required to confirm these results.

## Figures and Tables

**Table 1:** Demographic and clinical characteristics of individual subjects who contributed to plasma for healthy controls (HCs) and coronary artery disease (CAD) patients with various degrees of stenosis.

Variables ^a^		Discovery Set ^b^			Validation Set ^b^		
	Healthy Controls, *n* = 20	Coronary Artery Disease with Stenosis (%)		Healthy Controls, *n* = 40	Coronary Artery Disease with Stenosis (%)		
		≤50 *n* = 20	>50 *n* = 20		<30 *n* = 46	30~70 *n* = 47	>70 *n* = 79
Age (years)	34 ± 3.39	63.6 ± 9.36 **	63.4 ± 8.38 **	38.3 ± 10.19	62.93 ± 10.22 **	63.06 ± 9.86 **	62.43 ± 9.07 **
Gender							
Male, *n* (%)	14 (70.0)	14 (70.0)	14 (70.0)	24 (60.0)	31 (67.4)	33 (70.2)	60 (76.0)
Drinker, *n* (%)	6 (30.0)	6 (30.0)	6 (30.0)	16 (40.0)	15 (32.6)	14 (29.8)	19 (24.0)
Used to smoke, *n* (%)	0 (0)	9 (45.0) **	5 (25.0)	1 (2.5)	15 (32.6) **	8 (17.0)	18 (22.8) **
Current smoker, *n* (%)	6 (30.0)	1 (5.0)	5 (25.0)	13 (32.5)	4 (8.6) **	7 (14.8)	27 (34.1)
ESRD, *n* (%)	0 (0)	1 (5)	7 (35) *	0 (0)	1 (2.1)	2 (4.2)	13 (16.4) *
Hypertension, *n* (%)	1 (5.0)	14 (70.0) **	14 (70.0) **	2 (5.0)	30 (65.2) **	38 (80.8) **	49 (62.0) **
Lipid-lowering agents, *n* (%)	0 (0)	6 (30.0) **	11 (55.0) **	0 (0)	11 (23.9) **	15 (31.9) **	56 (70.8) **
Blood tests							
T-CHOL (mg/dL)	143.06 ± 30.56	143.23 ± 32.25	119.86 ± 43.53	158.19 ± 34.7	145.49 ± 35.92	139.42 ± 28.6 *	140.98 ± 45.98
HDL (mg/dL)	45.62 ± 13.06	48.08 ± 12.99	31.42 ± 9.57 *	50.25 ± 13.65	45.33 ± 12.88	48.81 ± 17.82	39.09 ± 12.26 **
LDL (mg/dL)	80.25 ± 37.04	81.29 ± 28.67	77.57 ± 34.21	92.97 ± 36.24	86.33 ± 30.95	79.04 ± 30.95	89.29 ± 38.69
TGs (mg/dL)	86.37 ± 30.5	82.55 ± 33	89.4 ± 34.8	76.97 ± 26.62	116.58 ± 118.17	103.88 ± 114.25	141.63 ± 162.55 *
PCr (mg/dL)	0.735 ± 0.122	0.935 ± 0.480	1.09 ± 0.453 *	0.753 ± 0.302	0.946 ± 0.365 *	0.917 ± 0.28	1.171 ± 0.884 *
HNE-protein adduct (μg/mL)	-	-	-	1.012 ± 0.079	1.054 ± 0.157	1.052 ± 0.105	1.116 ± 0.126 **

^a^ ESRD, end-stage renal disease; PCr, plasma creatinine; HNE-protein adduct, HNE-modified protein adduct; T-CHOL, total cholesterol (with a cutoff of <200 mg/dL); HDL, high-density lipoprotein (with a cutoff of >40 mg/dL); LDL, low-density lipoprotein (with a cutoff of <160 mg/dL); TGs, triglycerides (with a cutoff of <150 mg/dL); all were measured with a point of care testing (POCT) machine from Skyla (HCT, Taiwan, China) and Skyla Hi^@^ Lipid Panel Reagent Kit. ^b^ CAD, coronary artery disease. The *p*-values were determined using a *t*-test for continuous variables and a Chi-squared test for categorical variables. * *p* < 0.05, ** *p* < 0.01.

**Table 2:** Identification of novel types of 4-hydroxy-2-nonenal (HNE) modifications of apolipoprotein A-I (ApoA-I) in healthy controls (HCs) and coronary artery disease (CAD) patients with various levels of stenosis (≤50% and >50%).

Protein ^a^	Modified Peptide	Position ^b^	Obs. ^c^	Calc. ^d^	dM ^e^	dM ^f^	Sequence Found with Modification		
							HC	Coronary Artery Disease with Stenosis (%)	
								≤50	>50
ApoA-I	VSFLSALEEYTK(+138.10)	251~262	1523.812	1523.815	0.003	−7.5	-	+	+
ApoA-I	L(+138.10)L(+138.10)DNWDSVTSTFSK	70~83	1887.986	1887.985	−0.001	−0.34	-	+	-
ApoA-I	DYVSQ(+156.12)FEGSALGK	52~64	1555.770	1555.789	0.019	−8.6	+	+	+

^a^ ApoA-I, Apolipoprotein A-I. ^b^ Amino acid positions of the first and last residues in the accession number. ^c^ Obs.: observed mass of the modified peptides. ^d^ Calc.: calculated mass of the modified peptides. ^e^ dM (Obs.-Calc.): mass accuracy. ^f^ Modified peptide mass accuracy (ppm).

**Table 3:** Association between 4-hydroxy-2-nonenal-modified protein adducts (HNE-protein adducts) and autoantibody isotypes against unmodified and HNE-peptide adducts in coronary artery disease patients (CAD) with >30% compared to those with <30% stenosis.

	Coronary Artery Disease with Stenosis (%)				Multivariate Logistic Regression Model ^b^		Power
			<30 ^a^	>30			
	Cutoff		*n* = 86	*n* = 126	ORs (95% CI)	*p*-Value	
HNE-protein adduct	≤	1.02	47	36	Ref.	0.020	0.938
	>	1.02	39	90	2.208 (1.131, 4.311)		
IgG anti-ApoA-I^251–262^	>	4.49	24	22	Ref.	0.106	0.891
	≤	4.49	62	104	1.912 (0.870, 4.201)		
IgG anti-ApoA-I^251–262^ HNE	>	3.27	30	28	Ref.	0.202	0.542
	≤	3.27	56	98	1.612 (0.774, 3.357)		
IgG anti-ApoA-I^70–83^	>	2.91	58	65	Ref.	0.018	0.623
	≤	2.91	28	61	2.277 (1.149, 4.511)		
IgG anti-ApoA-I^70–83^ HNE	>	1.06	61	70	Ref.	0.026	0.665
	≤	1.06	25	56	2.190 (1.096, 4.374)		
IgM anti-ApoA-I^251–262^	>	0.50	51	50	Ref.	0.054	0.715
	≤	0.50	35	76	1.888 (0.988, 3.608)		
IgM anti-ApoA-I^251–262^ HNE	>	0.30	41	34	Ref.	0.035	0.881
	≤	0.30	45	92	2.046 (1.051, 3.984)		
IgM anti-ApoA-I^70–83^	>	0.59	45	38	Ref.	0.016	0.551
	≤	0.59	41	88	2.316 (1.172, 4.579)		
IgM anti-ApoA-I^70–83^ HNE	>	0.18	57	60	Ref.	0.316	0.372
	≤	0.18	29	66	1.427 (0.712, 2.860)		

^a^ The sample size (*n* = 86) of CAD patients with <30% stenosis included both healthy controls (*n* = 40) and CAD patients with <30% stenosis (*n* = 46). ^b^ Adjusted by age, gender, creatinine, and high-density lipoprotein. OR, odds ratio; CI, confidence interval.

## Data Availability

Acquired MS/MS fragmentation data are available in ProteomeXchange through the PRIDE database with accession no. PXD041969.

## Supplementary Materials

The following supporting information can be downloaded at https://www.mdpi.com/article/10.3390/cimb46060374/s1, Figure S1: MS/MS spectrum of the 4-hydroxy-2-nonenal (HNE)-modified peptide; Figure S2: The measurement of the level of apolipoprotein A-I (ApoA-I); Figure S3: Correlation of autoantibody isotypes against unmodified and 4-hydroxy-2-nonenal (HNE)-modified apolipoprotein A-I (ApoA-I) peptide adducts, HNE-modified protein adduct, and blood tests in coronary artery disease (CAD) patients with varying degrees of stenosis (30%, 30–70%, and >70%); Table S1: 4-hydroxy-2-nonenal (HNE)-modified sequences and sites of apolipoprotein A-I (ApoA-I); Table S2: Sample size estimation.

## Abbreviations

1D SDS-PAGE, one-dimensional sodium dodecylsulfate–polyacrylamide gel electrophoresis; ApoA-I, apolipoprotein A-I; AUROC, area under the receiver operating characteristic curve; CAD, coronary artery disease; CBB, Coomassie brilliant blue; CI, confidence interval; PCr, plasma creatinine; CVD, cardiovascular disease; ELISA, enzyme-linked immunosorbent assay; HC, healthy control; HDL, high-density lipoprotein; Ig, immunoglobulin; IP, immunoprecipitation; HNE, 4-hydroxy-2-nonenal; LDL, low-density lipoprotein; NAA, natural autoantibody; nano-LC-MS/MS, nano-liquid chromatography-tandem mass spectroscopy; OR, odds ratio; OSE, oxidation-specific epitope; Ox, oxidized; PTM, post-translational modification; ROC, receiver operating characteristic; T-CHOL, total cholesterol; TGs, triglycerides.
