# Supplementary material for: Low Levels of IgM Recognizing 4-Hydroxy-2-Nonenal-Modified Apolipoprotein A-I Peptide and Its Association with the Severity of Coronary Artery Disease in Taiwanese Patients
Source: Curr Issues Mol Biol. 2024 Jun 20;46(6):6267–83. doi: 10.3390/cimb46060374 (PMC11202877; doi:10.3390/cimb46060374)
Supplement: Supplementary file 1 [file cimb-46-00374-s001.zip › 0 Supplementary Information ApoA1-HNE 20230729 v1.1.docx]

**Supplementary information**

1. **Supplementary methods**

**1.1. Measurement of plasma protein concentrations**

Plasma protein concentrations were measured using a PierceTM Coomassie Plus (Bradford) Assay Kit (Thermo Scientific, Waltham, MA, USA). In a 96-well plate with a reagent (200 μL), plasma samples (4 μL) and bovine serum albumin (BSA) standards were added and incubated for 10 min at 37 °C. The optical density (OD) was determined at 595 nm. Protein concentrations of samples were determined using the BSA standard range (0–1500 g/mL).

**1.2. One-dimensional sodium dodecylsulfate-polyacrylamide gel electrophoresis (1D SDS-PAGE) and in-gel digestion**

Plasma protein samples (50 μg) were run on a 10% 1D SDS-PAGE (Hoefer®, Holliston, MA, USA) gel. The entire gel was stained with a Coomassie brilliant blue (CBB) staining solution (Bio-Rad Laboratories, Hercules, CA, USA). Following the previous western blot results of ApoA-I (data not shown), a slice of gel was cut based on its molecular weight of 24–26 kDa. The gel piece was destained in a solution of 25 mM NH_4_HCO_3_ and 50% (v/v) acetonitrile (1:1) until no protein bands were visible. Gel slices were repeatedly destained using 25 mM NH_4_HCO_3_ and 50% (v/v) acetonitrile (1:1). After drying, the gel slices were incubated with 2% β-mercaptoethanol and 25 mM NH_4_HCO_3_ for 20 min at room temperature in the dark to reduce disulfide bonds in the proteins. In the cysteine alkylation process, 10% vinyl pyridine in 25 mM NH_4_HCO_3_ and 50% acetonitrile were added and incubated for 20 min. Gel slices were soaked in 25 mM NH_4_HCO_3_ for 10 min and dried in a Speed-Vac (Thermo Electron, Waltham, MA, USA) for 20 min. Subsequently, modified trypsin (100 ng, Promega, Mannheim, Germany) in 25 mM NH_4_HCO_3_ was added to the gel slices, and the mixture was incubated at 37 °C overnight after tryptic digestion. Tryptic peptides were collected and dried using the Speed-Vac. Tryptic peptides were stored at −20 °C for further analysis. Tryptic peptides were dissolved in 0.1% formic acid before use.

**1.3 Identification of 4-hydroxy-2-nonenal (HNE) modifications using nano-liquid chromatography-tandem mass spectrometry (nano-LC-MS/MS) and PEAKS 7 software**

Peptide mixtures were analyzed on a 75-μm I.D., 25-cm-long C18 BEH column (Waters, Milford, MA, USA) packed with 1.7-μm particles with a pore width of 130 Å and were separated using a segmented gradient in 30 min from 5% to 40% of solvent B (acetonitrile with 0.1% formic acid) at a flow rate of 300 nL/min and a column temperature of 35 °C. Solvent A was 0.1% formic acid in water. The mass spectrometer was operated in the data-dependant mode. Briefly, survey full-scan MS spectra were gained in the orbitrap (m/z 350~1600) with the resolution set to 60,000 at m/z 400 and an automatic acquire control (AGC) target of 10^6^. The 10 most intense ions were sequentially isolated for charge injection device (CID) MS/MS fragmentation and detection in the linear ion trap (with an AGC target of 7000) with previously selected ions dynamically excluded for 90 s. Ions with single and unrecognized charge states were also excluded.

The MS dataset of protein identification was analyzed by automated de novo sequencing conducted using PEAKS 7 software (Bioinformatics Solutions, Waterloo, Canada). The homology search was performed by comparing de novo sequence tags with the human protein sequence database with 157,433 entries (obtained from UniProt; http://www.uniprot.org/, 2016/11). PEAKS 7 uses an expectation-maximization (EM)-based algorithm for feature detection, deconvolution, and refinement. An optimization model for simultaneous feature matching and retention time alignment was used in the analysis.

HNE, a byproduct of lipid peroxidation reactions, can interact with amino acid residues in proteins to form either Michael or Schiff base adducts [1]. The leucine (L), lysine (K), histidine (H), glutamine (Q), cysteine (C), alanine (A), and arginine (R) of HNE-amino acid Michael adducts exhibit an increase in mass of 156 Da, respectively, in which the alkene bond in HNE interacts with particular amino acid residues in the protein. Moreover, the amino acid residues (CHKAL) of HNE-amino acid Schiff base adducts respectively exhibit a mass addition of 138 Da in which the aldehyde group of HNE attacks amino acid residues in the proteins [2-4]. HNE-modified peptide (HNE-peptide) sequences and sites were identified using the PeaksPTM module of PEAKS 7 software. Carbamidomethylation (C)/+57.02 Da was set as the fixed modification, and oxidation (M)/+15.99 Da was selected as the variable modification. The following HNE modifications were specified as variables: ① CHKRQAL/+156.11504 Da (Michael adduct), ② CHKAL/+138.10446 Da (Schiff-base adduct). The maximum missed cleavage was set to 2. The mass tolerance of precursor ions was set to 10 ppm, and the fragment ion tolerance with a monoisotopic mass was set to 0.6 Da. Using a false detection rate (FDR) of 1.0% and a significant score (−10lgP) for peptides of ≥22, proteins with a target-decoy database search were accepted if at least two peptides and two unique peptides were detected. Maximum variable post-translation modifications (PTMs) were set to 5. Furthermore, all modified MS spectra were manually identified in this study, and fragmented ions (from the nano-LC/MS/MS) were labeled as b, y, y-NH_3_, and b-H_2_O ions.

**1.4 Immunoprecipitation (IP)**

HNE-modified apolipoprotein A-I (HNE-ApoA-I) was immunoprecipitated from pooled and individual plasma samples. Two micrograms of a mouse anti-ApoA-I monoclonal antibody (H00000335-M01, Abnova, Taipei, Taiwan) were coupled to 2 mg of Protein A Sepharose™ CL-4B (GE Healthcare Bio-Sciences, Pittsburgh, PA, USA) in 400 μL of NET-2 buffer (50 mM Tris-HCl, 150 mM NaCl, and 0.5% Triton X100; at pH 7.4), then incubated with 200 μg of IgG-removal serum proteins and mixed gently for 2 h at 4 °C. Beads were washed three times with 1 mL of NET-2* buffer (50 mM Tris-HCl at pH 7.4, 150 mM NaCl, and 0.05% Triton X100) and centrifuged at 4000 rpm for 3 min. Finally, 5 μg immunoprecipitated ApoA-I was separated on 10% SDS-PAGE gels, and then HNE modification of ApoA-I was validated through Western blotting (WB) using a goat polyclonal anti-HNE antibody (MBS536107, MyBioSource, San Diego, CA, USA). Another duplicate protein gel stain used the CBB staining solution (Bio-Rad Laboratories) that served as a loading control.

**1.5 Western blotting (WB)**

WB used a 10% SDS-PAGE gel and 5 µg loaded amounts of plasma proteins. Proteins were separated through an appropriate SDS-PAGE procedure in Tris-glycine buffer (25 mM Tris, 192 mM glycine, and 0.1% w/v SDS; pH 8.3) and transferred onto a polyvinylidene difluoride (PVDF) membrane in transfer buffer (25 mM Tris base, 192 mM glycine, and 20% methanol; pH 8.0; GE Healthcare Life Sciences, Piscataway, NJ, USA). The membrane was blocked with protein-free blocking buffer (BF01-1L, Visual Protein, Taipei, Taiwan) with subsequent incubation with a mouse anti-ApoA-I monoclonal antibody (H00000335-M01, Abnova) or a goat polyclonal anti-HNE antibody (MBS536107, dilution 1:5000, MyBioSource) to detect HNE-modified proteins (HNE-proteins). After being washed, the membrane was incubated with horseradish peroxidase (HRP)-conjugated anti-mouse (sc-2055) or anti-goat (sc-2354) monoclonal IgG antibody (both diluted 1:10000; Santa Cruz Biotechnology) that served as the secondary antibody. Visualization of the protein bands used the Luminata^TM^ Forte Western HRP Substrate (Millipore). The visualized band intensity was digitized and counted using an ImageQuant 400^TM^ Imager (GE Healthcare Life Sciences) and ImageJ software (National Institutes of Health, Bethesda, MD, USA). Another duplicate protein gel stain used the CBB staining solution (Bio-Rad Laboratories) that served as a loading control.

**1.6 Measurement of HNE-protein adduct using an enzyme-linked immunosorbent assay (ELISA)**

We adsorbed 100 µL of serum samples (10 μg/mL) or reduced/HNE-modified BSA (HNE-BSA; STA-335, Cell Biolabs, San Diego, CA, USA) standards (0~10 μg/mL) on a flat-bottomed 96-well plate with subsequent incubation of the plate at 37 °C for 2 h. After washing the plate with phosphate-buffered saline (PBS) containing 0.05% Tween 20 (PBST) and blocking it with protein-free blocking buffer (BF01-1L, Visual Protein) at room temperature for 1 h, we added the HRP-conjugated goat anti-HNE antibody (MBS390040, dilution 1:5000, Cell Biolabs) and incubated samples and standards at 37 °C for 3 h. Subsequently, after washing the plate, we detected the bound antibody-HRP using the SureBlue Reserve^TM^ TMB Microwell Peroxidase Substrate (Kirkegaard & Perry Laboratories, Gaithersburg, MD, USA) after incubation for 30 min at room temperature. The color reaction was ended by 1 N HCl, and the absorbance was measured at 450 nm. The concentration of HNE-protein adducts in serum was measured by a standard HNE-BSA curve. The concentration of HNE-protein adducts is expressed as µg/mL. An experiment was repeated if the coefficient of variation (CV%) was calculated to be >20%.

**1.7 Measurement of autoantibodies against unmodified and HNE-peptides using an ELISA**

Peptides (1 mg/ml) were modified with 2 mM HNE in 0.1 M phosphate buffer (pH 7.4) at 37 °C for 3 h. HNE-peptide was dialyzed against PBS containing 1 mM EDTA, which was changed four times for 18 h at 4 °C. Diluted peptides (10 µg/mL) with PBS (pH 7.4) were absorbed onto a plate and then incubated overnight at 4 °C. After washing and blocking the plates, we incubated them with 100-fold-diluted plasma and PBS at 37 °C for 2 h. After washing the plates again, we used rabbit anti-human IgG-HRP (dilution 1:10000, Santa Cruz Biotechnology) or rabbit anti-human IgM-HRP (dilution 1:10000, Santa Cruz Biotechnology) to detect deposition of the autoantibodies directed to an unmodified or HNE- peptide at 37 °C for 1 h. The antibody-HRP was reacted with the SureBlue Reserve^TM^ TMB Microwell Peroxidase Substrate (Kirkegaard & Perry Laboratories) with subsequent incubation for 10 min at room temperature. The color reaction was ended with 1 N HCl, and the absorbance value was measured at 450-620 nm. An experiment was repeated if the CV% was calculated to be >20%.

1. Sayre, L.M.; Lin, D.; Yuan, Q.; Zhu, X.; Tang, X. Protein adducts generated from products of lipid oxidation: focus on HNE and one. *Drug Metab Rev* **2006**, *38*, 651-675, doi:10.1080/03602530600959508.

2. Zhao, J.; Chen, J.; Zhu, H.; Xiong, Y.L. Mass spectrometric evidence of malonaldehyde and 4-hydroxynonenal adductions to radical-scavenging soy peptides. *J Agric Food Chem* **2012**, *60*, 9727-9736, doi:10.1021/jf3026277.

3. Lv, L.; Lin, H.; Li, Z.; Yuan, F.; Gao, Q.; Ma, J. Effect of 4-hydroxy-2-nonenal treatment on the IgE binding capacity and structure of shrimp (Metapenaeus ensis) tropomyosin. *Food Chem* **2016**, *212*, 313-322, doi:10.1016/j.foodchem.2016.05.152.

4. Uchida, K. 4-Hydroxy-2-nonenal: a product and mediator of oxidative stress. *Prog Lipid Res* **2003**, *42*, 318-343.
