# Supplementary material for: Low Levels of IgM Recognizing 4-Hydroxy-2-Nonenal-Modified Apolipoprotein A-I Peptide and Its Association with the Severity of Coronary Artery Disease in Taiwanese Patients
Source: Curr Issues Mol Biol. 2024 Jun 20;46(6):6267–83. doi: 10.3390/cimb46060374 (PMC11202877; doi:10.3390/cimb46060374)
Supplement: Supplementary file 1 [file cimb-46-00374-s001.zip › 2 Legends of Supplementary Figures and Tables.docx]

**Legends of Supplementary Figures and Tables**

Supplementary Figure 1. (A) A representative MS/MS spectrum of the ^251^-VSFLSALEEYTK-^262^ peptide sequence in apolipoprotein A-I (ApoA-I) and the 4-hydroxy-2-nonenal (HNE)-modified peptide had a 138.10446-Da shift of the HNE Schiff base adduct at lysine (K) 262. (B) MS/MS spectrum of the ^70^-LLDNWDSVTSTFSK-^83^ peptide sequence in ApoA-I and the 4-hydroxy-2-nonenal (HNE)-modified peptide with a 138.10446-Da shift in the two residues (at leucine (L) 70 and L71). (C) MS/MS spectrum of the ^52^-DYVSQFEGSALGK-^64^ peptide sequence in ApoA-I and the HNE-modified peptide with an increased mass of 156.11504 Da with HNE Michael adduct at glutamine (Q) 56.

Supplementary Figure 2. Protein levels of apolipoprotein A-I (ApoA-I) in plasma were determined by Western blotting (WB) using an anti-ApoA-I monoclonal antibody. A 10% SDS-PAGE gel and 5 µg of plasma protein were used in WB for ApoA-I (left, upper panel). A duplicate gel was stained with Coomassie brilliant blue (CBB) as a loading control (left, bottom panel). The red arrow indicates the ApoA-I protein. From duplicate gel band images, average blot densitometric values were calculated (right panel).

Supplementary Figure 3. Correlation of autoantibody isotypes against unmodified and 4-hydroxy-2-nonenal (HNE)-modified apolipoprotein A-I (ApoA-I) peptide adducts, HNE-modified protein adduct, and blood tests in coronary artery disease (CAD) patients with varying degrees of stenosis (30%, 30%–70%, and >70%). (A) IgG anti-ApoA-I^251-262^. (B) IgG anti-ApoA-I^251-262^ HNE. (C) IgG anti-ApoA-I^70–83^. (D) IgG anti-ApoA-I^70–83^ HNE. (E) IgM anti-ApoA-I^251-262^. (F) IgM anti-ApoA-I^251-262^ HNE. (G) IgM anti-ApoA-I^70–83^. (H) IgG anti-ApoA-I^70–83^ HNE. (I) HNE-modified protein adduct.

Supplementary Table 1. 4-hydroxy-2-nonenal (HNE)-modified sequences and sites of apolipoprotein A-I (ApoA-I).

Supplementary Table 2. Sample size estimation.
