# Supplementary material for: Low Levels of IgM Recognizing 4-Hydroxy-2-Nonenal-Modified Apolipoprotein A-I Peptide and Its Association with the Severity of Coronary Artery Disease in Taiwanese Patients
Source: Curr Issues Mol Biol. 2024 Jun 20;46(6):6267–83. doi: 10.3390/cimb46060374 (PMC11202877; doi:10.3390/cimb46060374)
Supplement: Supplementary file 1 [file cimb-46-00374-s001.zip › 2 Supplementary Figure S3 Correlation v1.5 ALL.pdf]

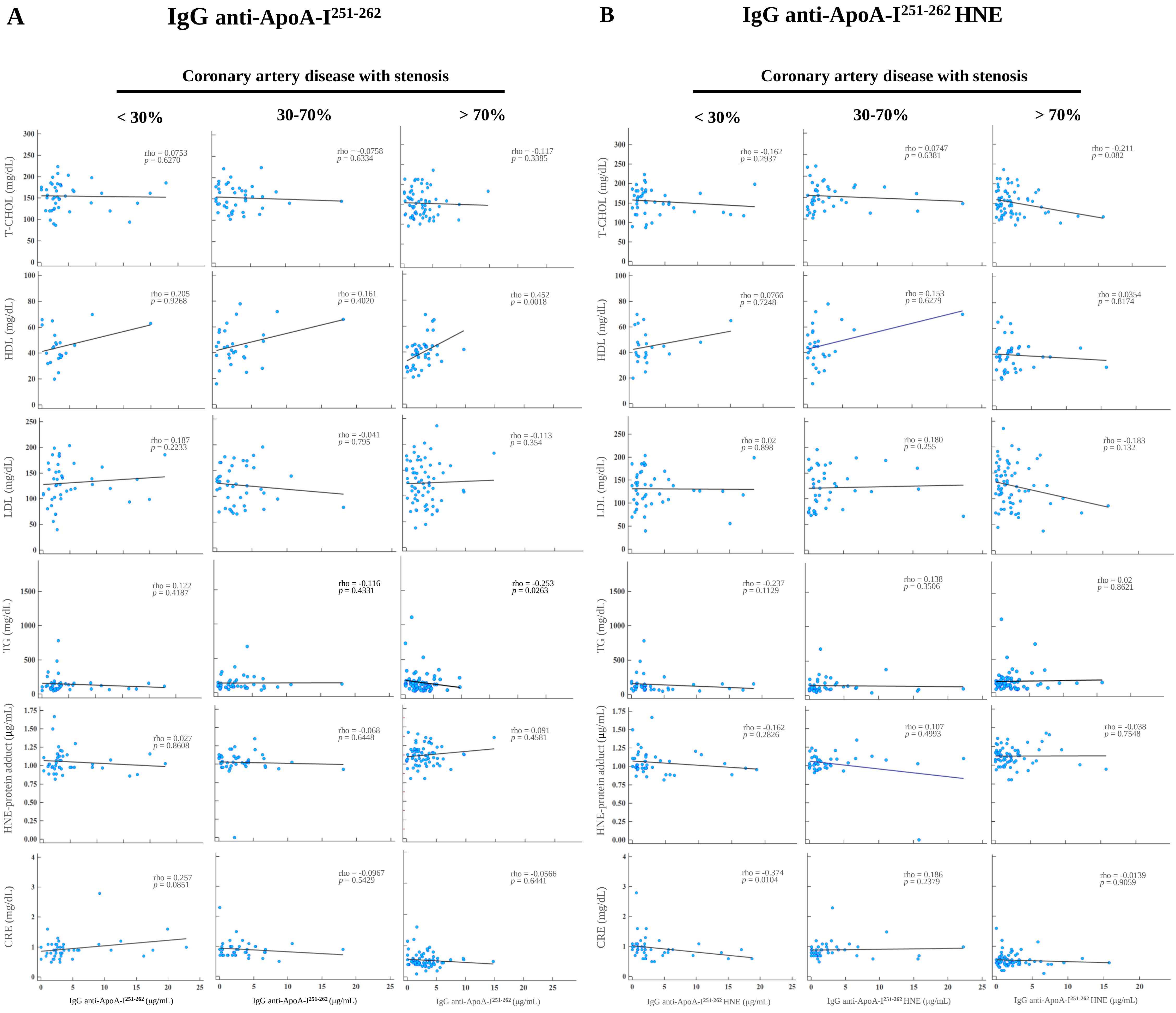
A
IgG anti-ApoA-I251-262
B
IgG anti-ApoA-I251-262 HNE
Coronary artery disease with stenosis
Coronary artery disease with stenosis
< 30%
30-70%
> 70%
< 30%
30-70%
> 70%
T-CHOL (mg/dL)
HDL (mg/dL)
LDL (mg/dL)
TG (mg/dL)
HNE-protein adduct (µg/mL)
CRE (mg/dL)
rho = 0.0753
p = 0.6270
rho = -0.0758
p = 0.6334
rho = -0.117
p = 0.3385
rho = -0.162
p = 0.2937
rho = 0.0747
p = 0.6381
rho = -0.211
p = 0.082
rho = 0.205
p = 0.9268
rho = 0.161
p = 0.4020
rho = 0.452
p = 0.0018
rho = 0.0766
p = 0.7248
rho = 0.153
p = 0.6279
rho = 0.0354
p = 0.8174
rho = 0.187
p = 0.2233
rho = -0.041
p = 0.795
rho = -0.113
p = 0.354
rho = 0.02
p = 0.898
rho = 0.180
p = 0.255
rho = -0.183
p = 0.132
rho = 0.122
p = 0.4187
rho = -0.116
p = 0.4331
rho = -0.253
p = 0.0263
rho = -0.237
p = 0.1129
rho = 0.138
p = 0.3506
rho = 0.02
p = 0.8621
rho = 0.027
p = 0.8608
rho = -0.068
p = 0.6448
rho = 0.091
p = 0.4581
rho = -0.162
p = 0.2826
rho = 0.107
p = 0.4993
rho = -0.038
p = 0.7548
rho = 0.257
p = 0.0851
rho = -0.0967
p = 0.5429
rho = -0.0566
p = 0.6441
rho = -0.374
p = 0.0104
rho = 0.186
p = 0.2379
rho = -0.0139
p = 0.9059
IgG anti-ApoA-I251-262 (µg/mL)
IgG anti-ApoA-I251-262 (µg/mL)
IgG anti-ApoA-I251-262 (µg/mL)
IgG anti-ApoA-I251-262 HNE (µg/mL)
IgG anti-ApoA-I251-262 HNE (µg/mL)
IgG anti-ApoA-I251-262 HNE (µg/mL)

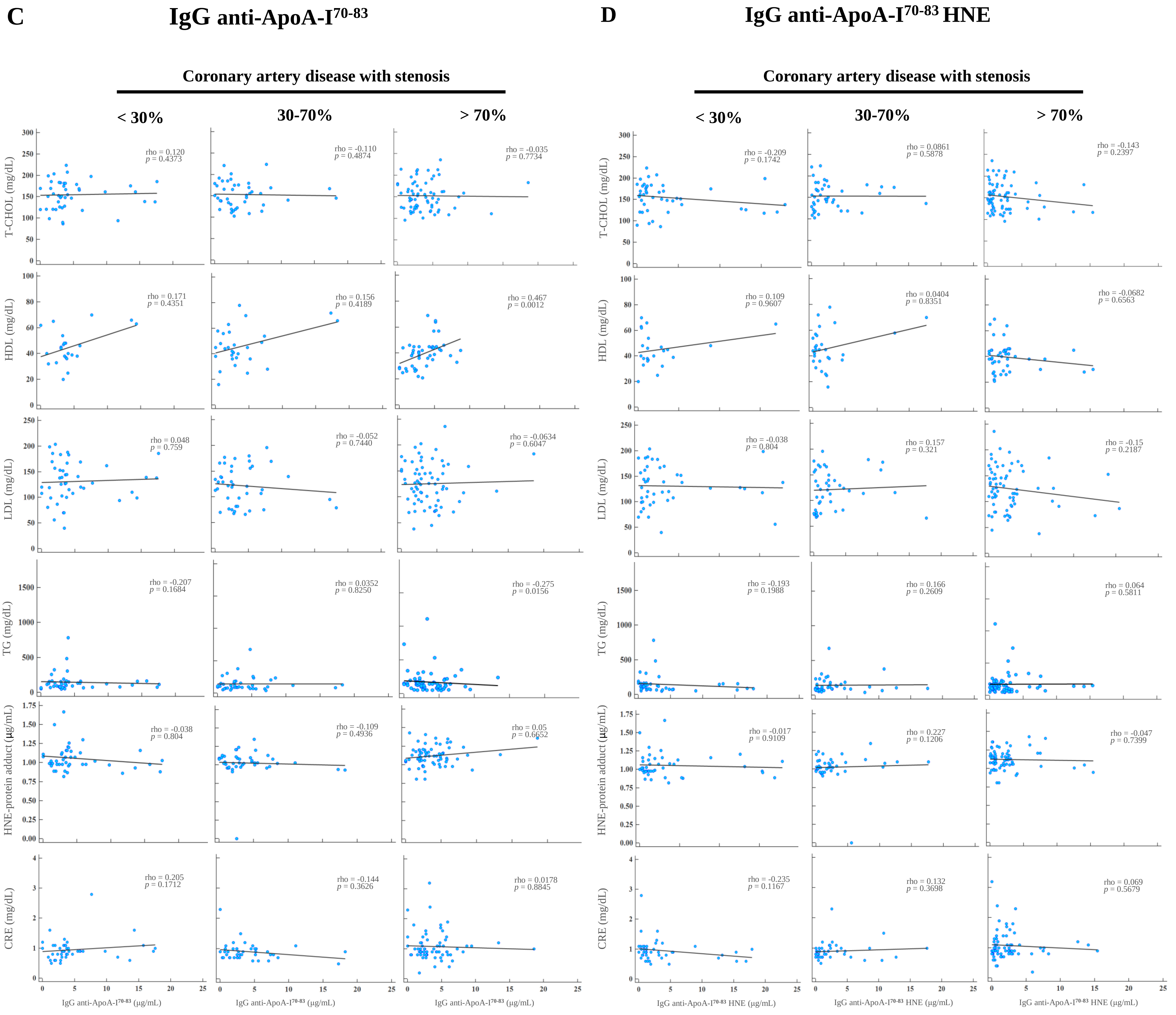
C
IgG anti-ApoA-I70-83
Coronary artery disease with stenosis
< 30%
30-70%
> 70%
T-CHOL (mg/dL)
rho = 0.120
p = 0.4373
rho = -0.110
p = 0.4874
rho = -0.035
p = 0.7734
HDL (mg/dL)
rho = 0.171
p = 0.4351
rho = 0.156
p = 0.4189
rho = 0.467
p = 0.0012
LDL (mg/dL)
rho = 0.048
p = 0.759
rho = -0.052
p = 0.7440
rho = -0.0634
p = 0.6047
TG (mg/dL)
rho = -0.207
p = 0.1684
rho = 0.0352
p = 0.8250
rho = -0.275
p = 0.0156
HNE-protein adduct (μg/mL)
rho = -0.038
p = 0.804
rho = -0.109
p = 0.4936
rho = 0.05
p = 0.6652
CRE (mg/dL)
rho = 0.205
p = 0.1712
rho = -0.144
p = 0.3626
rho = 0.0178
p = 0.8845
IgG anti-ApoA-I70-83 (μg/mL)
D
IgG anti-ApoA-I70-83 HNE
Coronary artery disease with stenosis
< 30%
30-70%
> 70%
T-CHOL (mg/dL)
rho = -0.209
p = 0.1742
rho = 0.0861
p = 0.5878
rho = -0.143
p = 0.2397
HDL (mg/dL)
rho = 0.109
p = 0.9607
rho = 0.0404
p = 0.8351
rho = -0.0682
p = 0.6563
LDL (mg/dL)
rho = -0.038
p = 0.804
rho = 0.157
p = 0.321
rho = -0.15
p = 0.2187
TG (mg/dL)
rho = -0.193
p = 0.1988
rho = 0.166
p = 0.2609
rho = 0.064
p = 0.5811
HNE-protein adduct (μg/mL)
rho = -0.017
p = 0.9109
rho = 0.227
p = 0.1206
rho = -0.047
p = 0.7399
CRE (mg/dL)
rho = -0.235
p = 0.1167
rho = 0.132
p = 0.3698
rho = 0.069
p = 0.5679
IgG anti-ApoA-I70-83 HNE (μg/mL)

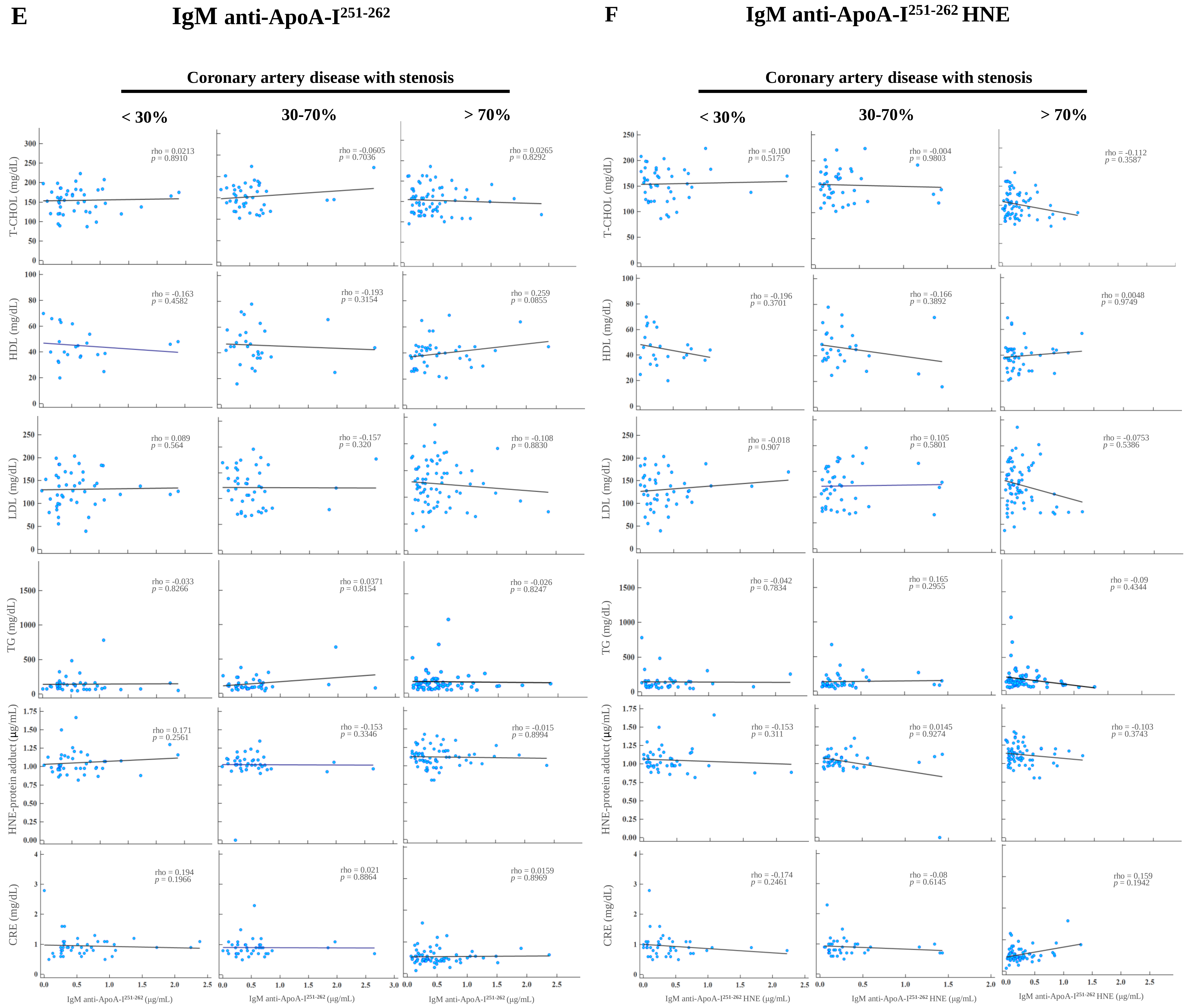
E
IgM anti-ApoA-I251-262
Coronary artery disease with stenosis
< 30%
30-70%
> 70%
T-CHOL (mg/dL)
HDL (mg/dL)
LDL (mg/dL)
TG (mg/dL)
HNE-protein adduct (μg/mL)
CRE (mg/dL)
rho = 0.0213
p = 0.8910
rho = -0.0605
p = 0.7036
rho = 0.0265
p = 0.8292
rho = -0.163
p = 0.4582
rho = -0.193
p = 0.3154
rho = 0.259
p = 0.0855
rho = 0.089
p = 0.564
rho = -0.157
p = 0.320
rho = -0.108
p = 0.8830
rho = -0.033
p = 0.8266
rho = 0.0371
p = 0.8154
rho = -0.026
p = 0.8247
rho = 0.171
p = 0.2561
rho = -0.153
p = 0.3346
rho = -0.015
p = 0.8994
rho = 0.194
p = 0.1966
rho = 0.021
p = 0.8864
rho = 0.0159
p = 0.8969
IgM anti-ApoA-I251-262 (μg/mL)
F
IgM anti-ApoA-I251-262 HNE
Coronary artery disease with stenosis
< 30%
30-70%
> 70%
T-CHOL (mg/dL)
HDL (mg/dL)
LDL (mg/dL)
TG (mg/dL)
HNE-protein adduct (μg/mL)
CRE (mg/dL)
rho = -0.100
p = 0.5175
rho = -0.004
p = 0.9803
rho = -0.112
p = 0.3587
rho = -0.196
p = 0.3701
rho = -0.166
p = 0.3892
rho = 0.0048
p = 0.9749
rho = -0.018
p = 0.907
rho = 0.105
p = 0.5801
rho = -0.0753
p = 0.5386
rho = -0.042
p = 0.7834
rho = 0.165
p = 0.2955
rho = -0.09
p = 0.4344
rho = -0.153
p = 0.311
rho = 0.0145
p = 0.9274
rho = -0.103
p = 0.3743
rho = -0.174
p = 0.2461
rho = -0.08
p = 0.6145
rho = 0.159
p = 0.1942
IgM anti-ApoA-I251-262 HNE (μg/mL)

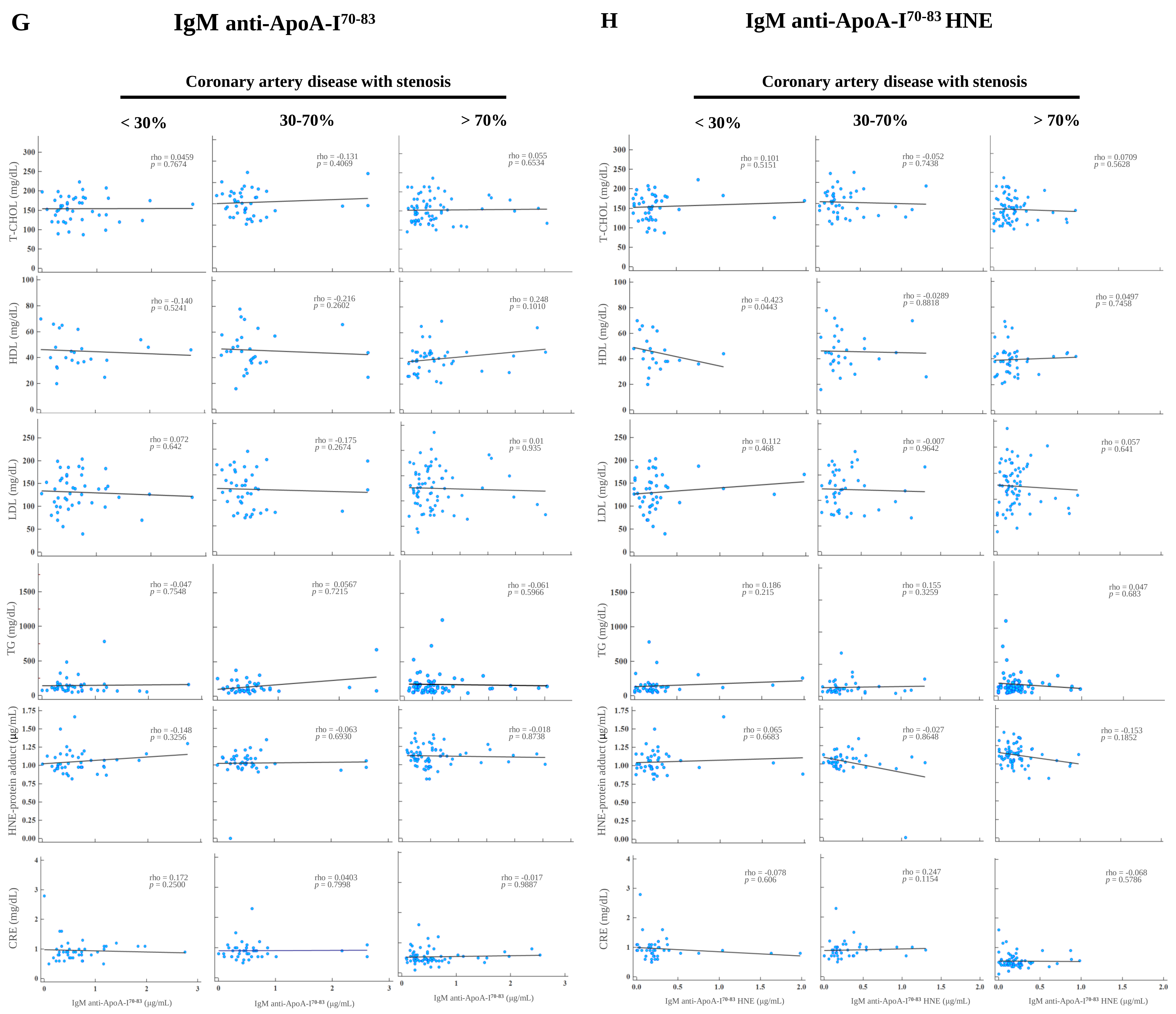
G
IgM anti-ApoA-I70-83
Coronary artery disease with stenosis
< 30%
30-70%
> 70%
T-CHOL (mg/dL)
rho = 0.0459
p = 0.7674
rho = -0.131
p = 0.4069
rho = 0.055
p = 0.6534
HDL (mg/dL)
rho = -0.140
p = 0.5241
rho = -0.216
p = 0.2602
rho = 0.248
p = 0.1010
LDL (mg/dL)
rho = 0.072
p = 0.642
rho = -0.175
p = 0.2674
rho = 0.01
p = 0.935
TG (mg/dL)
rho = -0.047
p = 0.7548
rho = 0.0567
p = 0.7215
rho = -0.061
p = 0.5966
HNE-protein adduct (μg/mL)
rho = -0.148
p = 0.3256
rho = -0.063
p = 0.6930
rho = -0.018
p = 0.8738
CRE (mg/dL)
rho = 0.172
p = 0.2500
rho = 0.0403
p = 0.7998
rho = -0.017
p = 0.9887
IgM anti-ApoA-I70-83 (μg/mL)
H
IgM anti-ApoA-I70-83 HNE
Coronary artery disease with stenosis
< 30%
30-70%
> 70%
T-CHOL (mg/dL)
rho = 0.101
p = 0.5151
rho = -0.052
p = 0.7438
rho = 0.0709
p = 0.5628
HDL (mg/dL)
rho = -0.423
p = 0.0443
rho = -0.0289
p = 0.8818
rho = 0.0497
p = 0.7458
LDL (mg/dL)
rho = 0.112
p = 0.468
rho = -0.007
p = 0.9642
rho = 0.057
p = 0.641
TG (mg/dL)
rho = 0.186
p = 0.215
rho = 0.155
p = 0.3259
rho = 0.047
p = 0.683
HNE-protein adduct (μg/mL)
rho = 0.065
p = 0.6683
rho = -0.027
p = 0.8648
rho = -0.153
p = 0.1852
CRE (mg/dL)
rho = -0.078
p = 0.606
rho = 0.247
p = 0.1154
rho = -0.068
p = 0.5786
IgM anti-ApoA-I70-83 HNE (μg/mL)

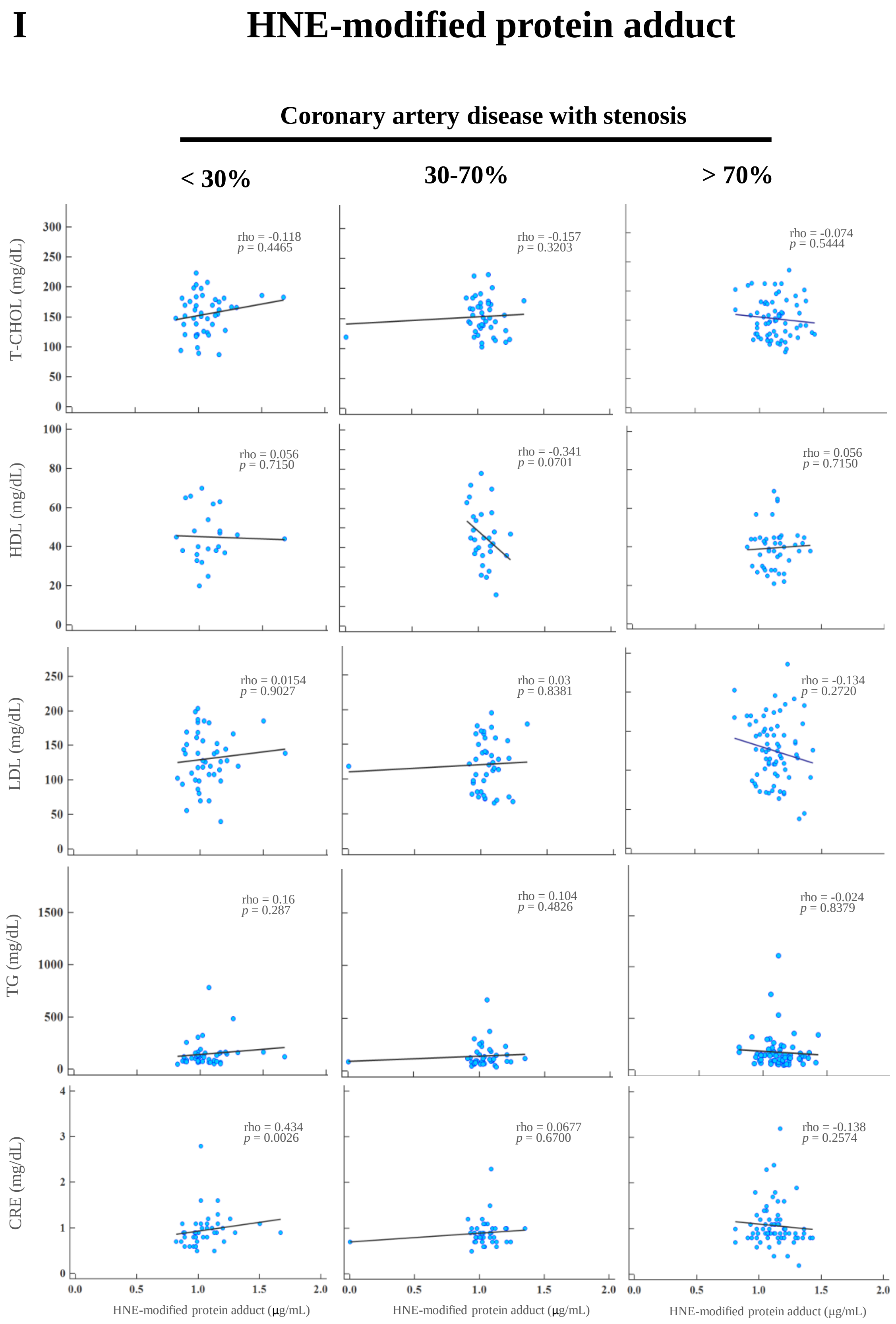
I
HNE-modified protein adduct
Coronary artery disease with stenosis
< 30%
30-70%
> 70%
T-CHOL (mg/dL)
HDL (mg/dL)
LDL (mg/dL)
TG (mg/dL)
CRE (mg/dL)
rho = -0.118
p = 0.4465
rho = -0.157
p = 0.3203
rho = -0.074
p = 0.5444
rho = 0.056
p = 0.7150
rho = -0.341
p = 0.0701
rho = 0.056
p = 0.7150
rho = 0.0154
p = 0.9027
rho = 0.03
p = 0.8381
rho = -0.134
p = 0.2720
rho = 0.16
p = 0.287
rho = 0.104
p = 0.4826
rho = -0.024
p = 0.8379
rho = 0.434
p = 0.0026
rho = 0.0677
p = 0.6700
rho = -0.138
p = 0.2574
HNE-modified protein adduct (μg/mL)
HNE-modified protein adduct (μg/mL)
HNE-modified protein adduct (μg/mL)
