# Supplementary material for: Low Levels of IgM Recognizing 4-Hydroxy-2-Nonenal-Modified Apolipoprotein A-I Peptide and Its Association with the Severity of Coronary Artery Disease in Taiwanese Patients
Source: Curr Issues Mol Biol. 2024 Jun 20;46(6):6267–83. doi: 10.3390/cimb46060374 (PMC11202877; doi:10.3390/cimb46060374)
Supplement: Supplementary file 1 [file cimb-46-00374-s001.zip › 2 Supplementary Table S2.docx]

We collected only 272 plasma samples from September 1, 2018, to October 31, 2019, and divided them into the discovery set (60 samples) and the validation set (212 samples). The validation set included 40 HCs, 46 CAD patients with <30% stenosis, 47 CAD patients with between 30%-70% stenosis, and 79 CAD patients with >70% stenosis. The sample size (*n* = 86) of CAD patients with <30% stenosis included both HCs (*n* = 40) and CAD patients with <30% stenosis (*n* = 46). We calculated the sample size using G*Power version 3.1 based on the mean and standard deviation (SD) of CAD patients with <30% stenosis and CAD patients with >30% stenosis. The sample sizes were analyzed below. The limited sample size is a problem. Thus, power is needed to evaluate. The power was analyzed below.

|  | **HNE-protein adduct** | **IgG anti-APOA-I^251-262^** | **IgG anti-APOA-I^251-262^ HNE** | **IgG anti-APOA-I^70-83^** | **IgG anti-APOA-I^70-83^ HNE** | **IgM anti-APOA-I^251-262^** | **IgM anti-APOA-I^251-262^ HNE** | **IgM anti-APOA-I^70-83^** | **IgM anti-APOA-I^70-83^ HNE** |
| --- | --- | --- | --- | --- | --- | --- | --- | --- | --- |
| <30% stenosis, n = 86 | | |  |  |  |  |  |  |  |
| Mean | 1.035 | 5.290 | 3.878 | 5.445 | 3.955 | 0.691 | 0.419 | 0.737 | 0.314 |
| SD | 0.130 | 5.478 | 4.263 | 4.520 | 4.622 | 0.463 | 0.379 | 0.470 | 0.308 |
| >30% stenosis, n = 126 | | |  |  |  |  |  |  |  |
| Mean | 1.092 | 3.461 | 2.876 | 4.312 | 2.743 | 0.540 | 0.281 | 0.604 | 0.262 |
| SD | 0.119 | 2.992 | 3.654 | 3.444 | 3.356 | 0.484 | 0.293 | 0.569 | 0.234 |
| Sample size estimation | | |  |  |  |  |  |  |  |
| α= 0.05, β = 0.1 | |  |  |  |  |  |  |  |  |
| <30%  samples | 73 | 89 | 238 | 191 | 169 | 149 | 92 | 234 | 419 |
| >30%  samples | 107 | 131 | 348 | 279 | 247 | 219 | 134 | 342 | 613 |
| α= 0.05, β = 0.2 | |  |  |  |  |  |  |  |  |
| <30%  samples | 53 | 64 | 172 | 138 | 122 | 108 | 67 | 169 | 303 |
| >30%  samples | 77 | 94 | 252 | 202 | 178 | 157 | 97 | 247 | 443 |
| Power | 0.938 | 0.891 | 0.542 | 0.623 | 0.665 | 0.715 | 0.881 | 0.551 | 0.372 |
