# Supplementary figures and images for: Low Levels of IgM Recognizing 4-Hydroxy-2-Nonenal-Modified Apolipoprotein A-I Peptide and Its Association with the Severity of Coronary Artery Disease in Taiwanese Patients
Source: Curr Issues Mol Biol. 2024 Jun 20;46(6):6267–83. doi: 10.3390/cimb46060374 (PMC11202877; doi:10.3390/cimb46060374)

**A**      **ApoA-I<sup>251-262</sup>**  
**VSFLSALEEYTK(+138.10)**

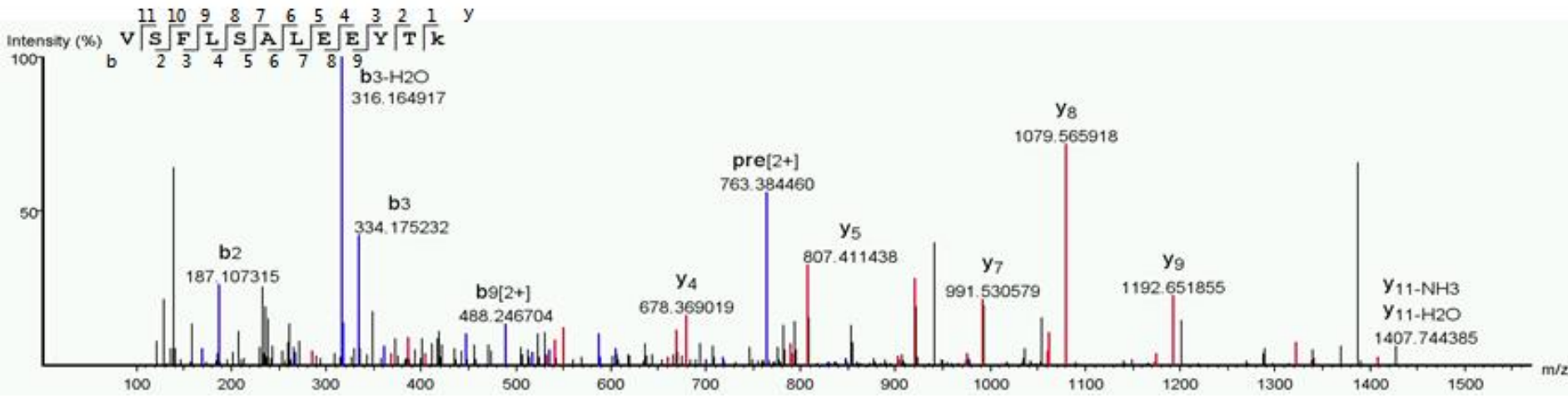

**B**      **ApoA-I<sup>70-83</sup>**  
**L(+138.10)L(+138.10)DNWDSVTSTFSK**

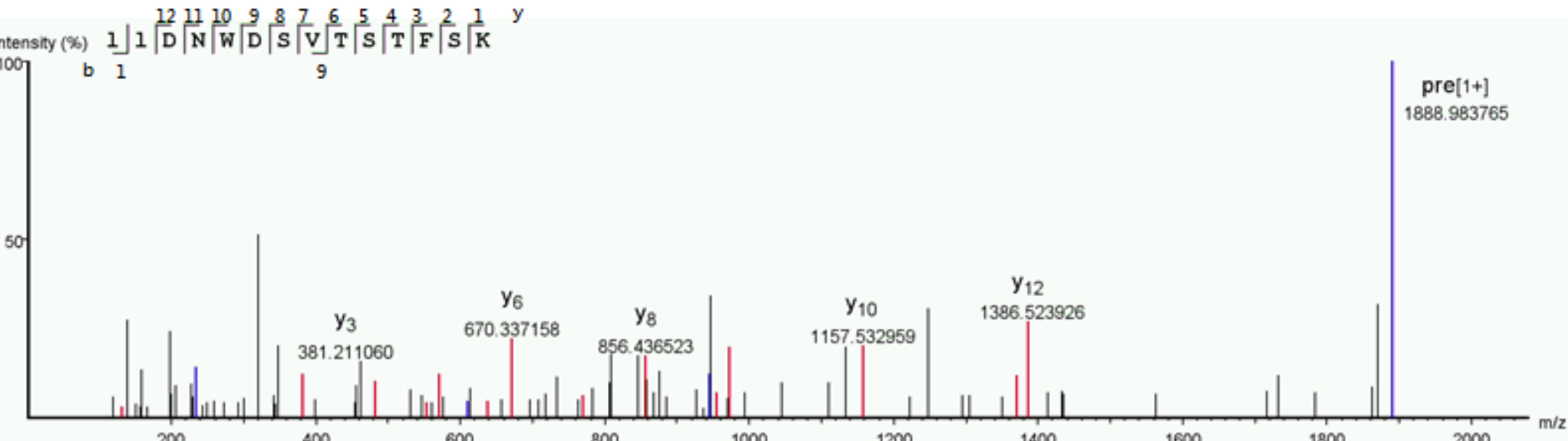

**C**      **ApoA-I<sup>52-64</sup>**  
**DYVSQ(+156.12)FEGSALGK**

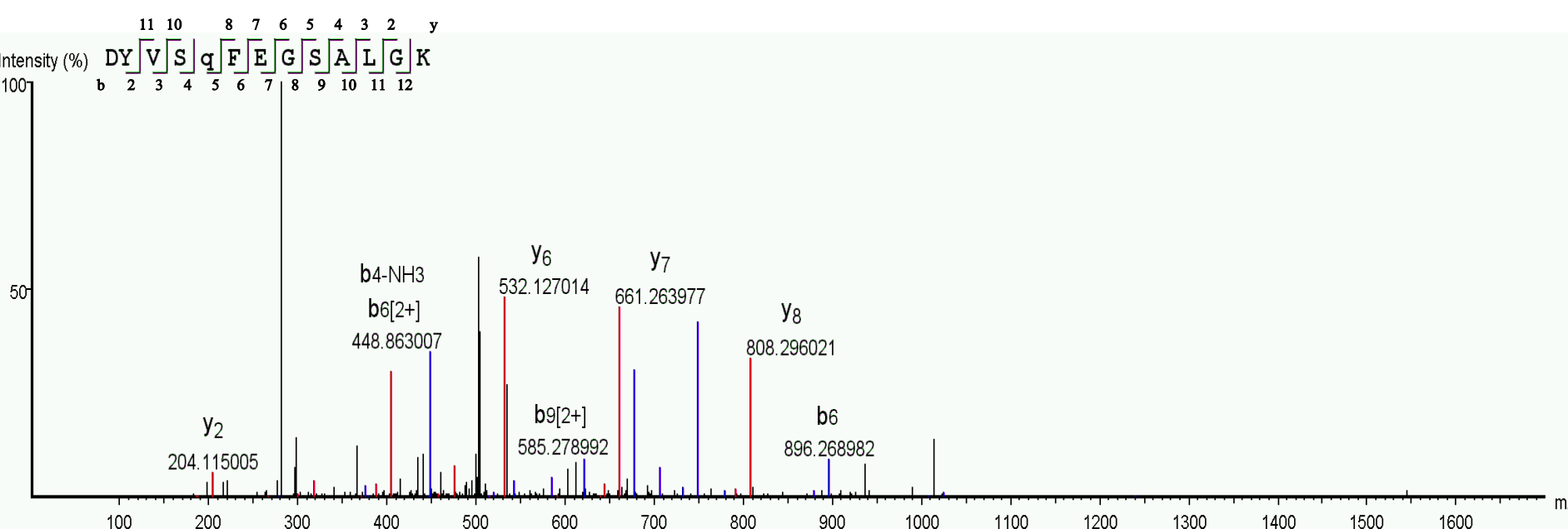

Supplement: Supplementary file 1 [file cimb-46-00374-s001.zip › 2 Supplementary Figure S1 ApoA1 HNE v1.1.pdf]

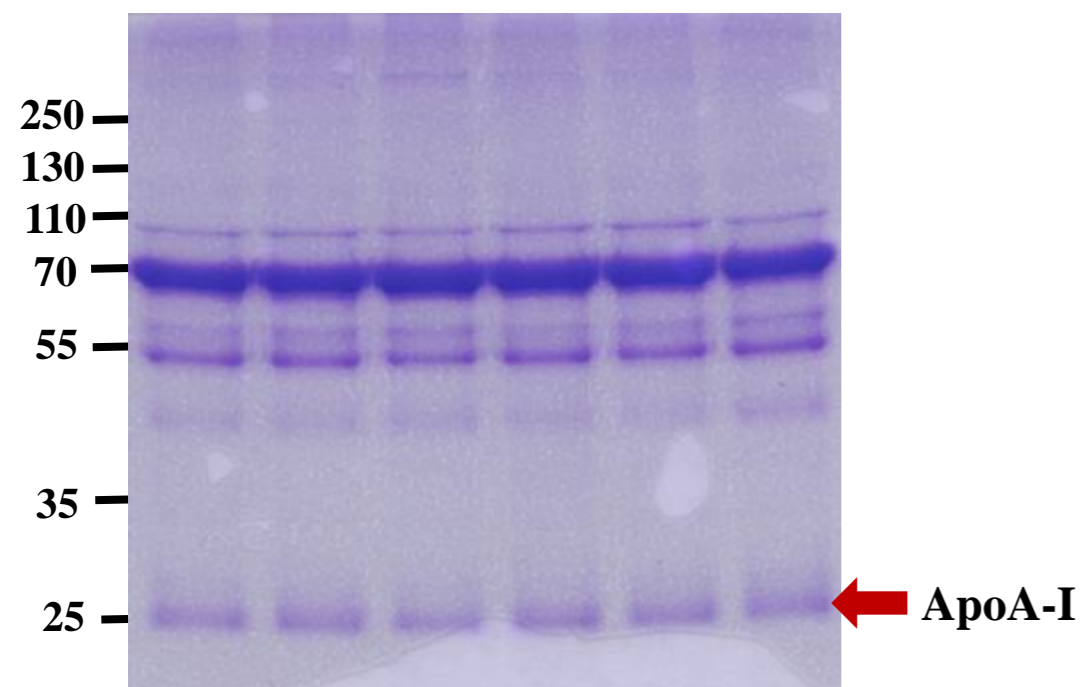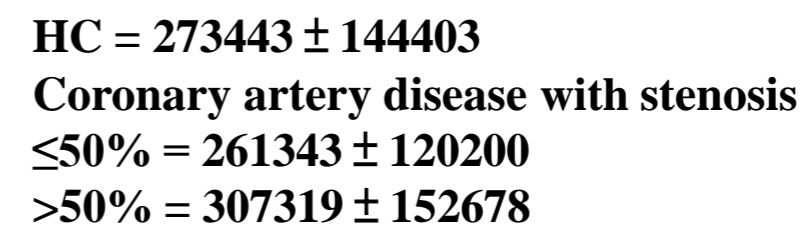

Supplement: Supplementary file 1 [file cimb-46-00374-s001.zip › 2 Supplementary Figure S2 ApoA-1 WB v1.3.pdf]
